# Systems modeling predicts that mitochondria ER contact sites regulate the postsynaptic energy landscape

**DOI:** 10.1038/s41540-021-00185-7

**Published:** 2021-06-02

**Authors:** A. Leung, D. Ohadi, G. Pekkurnaz, P. Rangamani

**Affiliations:** 1 Chemical Engineering Graduate Program, University of California San Diego, La Jolla, CA USA; 2 Department of Mechanical and Aerospace Engineering, University of California San Diego, La Jolla, CA USA; 3 Neurobiology Section, Division of Biological Sciences, University of California San Diego, La Jolla, CA USA

**Keywords:** Differential equations, Bioenergetics

## Abstract

Spatiotemporal compartmentation of calcium dynamics is critical for neuronal function, particularly in postsynaptic spines. This exquisite level of Ca^2+^ compartmentalization is achieved through the storage and release of Ca^2+^ from various intracellular organelles particularly the endoplasmic reticulum (ER) and the mitochondria. Mitochondria and ER are established storage organelles controlling Ca^2+^ dynamics in neurons. Mitochondria also generate a majority of energy used within postsynaptic spines to support the downstream events associated with neuronal stimulus. Recently, high resolution microscopy has unveiled direct contact sites between the ER and the mitochondria (MERCs), which directly channel Ca^2+^ release from the ER into the mitochondrial membrane. In this study, we develop a computational 3D reaction-diffusion model to investigate the role of MERCs in regulating Ca^2+^ and ATP dynamics. This spatiotemporal model accounts for Ca^2+^ oscillations initiated by glutamate stimulus of metabotropic and ionotropic glutamate receptors and Ca^2+^ changes in four different compartments: cytosol, ER, mitochondria, and the MERC microdomain. Our simulations predict that the organization of these organelles and inter-organellar contact sites play a key role in modulating Ca^2+^ and ATP dynamics.

We further show that the crosstalk between geometry (mitochondria and MERC) and metabolic parameters (cytosolic ATP hydrolysis, ATP generation) influences the neuronal energy state. Our findings shed light on the importance of organelle interactions in predicting Ca^2+^ dynamics in synaptic signaling. Overall, our model predicts that a combination of MERC linkage and mitochondria size is necessary for optimal ATP production in the cytosol.

## Introduction

Compartmentalized calcium handling in postsynaptic structures underlies synaptic communication and controls synaptic plasticity, which is the bidirectional modulation of synaptic strength that is thought to underlie learning and memory formation^1^. In excitatory neurons during synaptic transmission, glutamate released from the presynaptic bouton leads to a localized increase in calcium at the postsynaptic dendrite that is critical for the induction of synaptic plasticity^2,3^. At the postsynaptic site, small bulbous protrusions called dendritic spines act as biochemical computation units that regulate the duration and spread of postsynaptic calcium fluxes produced by glutamatergic neurotransmission^4^. The temporal dynamics of calcium, and the coupling to downstream signaling pathways in dendritic spines depend on many factors, including the nature of stimulus as well as the positioning of the calcium storage organelles, the endoplasmic reticulum (ER), and the mitochondria^5,6^. A continuous tubular network of ER spreads throughout the dendrites and extends into the spines either as a simple smooth tubule or a spine apparatus^7–9^.

**Fig. 1:**
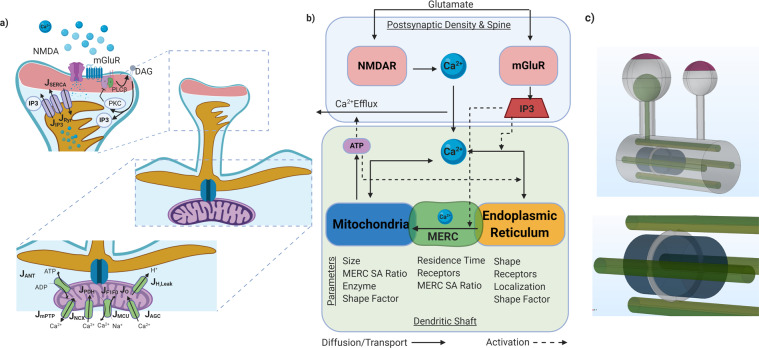
Schematic of the model architecture and fluxes in each compartment. **a** Glutamatergic signaling in a postsynaptic spine leads to an influx of Ca^2+^ from the extracellular space and from the endoplasmic reticulum. Dynamics of Ca^2+^ have a direct impact on mitochondrial function that alters ATP production^27,30^. The main fluxes involved in the model are shown here and the mathematical descriptions are given in Supplementary Table 2. **b** The main compartments and investigated parameters in the model are shown in this schematic. In this figure, solid lines represent diffusive or transport fluxes. Dashed lines represent an activation of fluxes. **c** Model geometry for a dendritic spine developed using COMSOL Multiphysics. Two spines, one with and one with the spine apparatus are connected by a dendritic shaft containing the ER and mitochondria. PSD, Purple; ER, Green; MERC, Gray; Mitochondria, Blue. SA Ratio refers to the ratio of the surface area of MERCs to total mitochondria surface area (**a** and **b**) were created using biorender.com.

Mitochondria of various lengths occupy a major portion of dendritic branches and associate closely with the ER particularly at the dendritic base of spines^10–12^. The intimate contact between mitochondria and ER along dendrites allows for a functional inter-organellar coupling and plays a central role in the regulation of the postsynaptic calcium dynamics (Fig. 1a). However, it must be noted that the distribution of both mitochondria and ER throughout spines is transient and heterogeneous^13^. Only 20% of spines contain ER and both ER and mitochondria cluster around actively potentiated spines^14^. Dysregulation of mitochondria and ER coupling have been demonstrated in neurodegenerative diseases such as Alzheimer’s^15–17^ and Parkinson’s^17,18^, although the underlying mechanisms are yet to be elucidated.

Physical contact sites between mitochondria and ER (Mitochondria-ER Contact Sites, or MERCs) were first observed by electron microscopy, then confirmed by dimerization-dependent fluorescent proteins^19,20^. MERCs provide a direct avenue for calcium transfer from the ER to the mitochondria^21^. Because the concentration of ER calcium is several orders of magnitude higher than cytosolic calcium, the existence of these contact sites can result in a direct conduit for increasing mitochondrial calcium concentration. These MERCs have been observed to be 2–20% of a mitochondria’s surface area^19^. MERCs are also essential for mitochondrial functions including Adenine triphosphate (ATP) production^22^. While the exact mechanism for the formation of these sites is unclear, there is evidence that binding proteins link via calcium channels on the surface of the respective organelle^20,23^. The role of organelles and MERC in calcium dynamics of dendritic spines is not yet fully understood.

Computational modeling has played a pivotal role in providing insight into calcium dynamics in neurons^24–26^. Bertram et al.^27^, Han et al.^28^, Wacquier et al.^29^, and others have created models addressing the role of calcium in mitochondria. Bertram et al.^27^ simplified a mathematical model for mitochondrial calcium dynamics in pancreatic beta cells originally developed by Magnus et al.^30^ to explain experimentally derived results^31^ in which calcium increases NADH under low glucose and reduces NADH in high glucose. Han et al.^28^ explicitly modeled the role MERC in a pancreatic beta-cell in a compartmental model for the case of a healthy and diabetic cells and predicted a connection between obesity, MERCs, and calcium dynamics. Qi et al. similarly modeled mitochondrial-ER calcium flux into the mitochondria as a function of distance between IP_3_ receptor (IP_3_R) and Mitochondrial Calcium Uniporter (MCU) to predict an optimal ER-to-mitochondria distance for the regulation of mitochondrial calcium^32^. However, these models are not specific to neurons and the unique circumstances of a dendritic spine. Previously, we and others have shown that spatial modeling of signaling pathways can provide insight into how cell shape and organelle organization can affect the spatiotemporal dynamics of second messengers and signaling cascades^33–38^. Although spatial models for neuronal calcium signaling exist^26,39,40^, these do not focus on the metabolic consequences of calcium dynamics and do not incorporate mitochondria.

In this study, we investigated the role of the ER, mitochondria, and MERC in modulating the spatiotemporal dynamics of calcium and localized ATP production in postsynaptic spines using computational modeling. We combined key cascades and parameters of glutamatergic receptor^29,41^, mitochondria^27^, and neuronal calcium signaling models^26,39^ to generate a spatial model in dendrites incorporating mitochondria and MERC in calcium signaling and ATP generation (Fig. 1). While calcium models are prevalent in the neuron literature, conversion to spatial properties and dendritic spine geometries presents a unique challenge. We specifically sought to answer the following questions: What are the spatiotemporal dynamics of Ca^2+^ and Inositol 1,4,5-trisphosphate (IP_3_), in response to a glutamate stimulus, in the spine head, spine shaft, and the mitochondria? How does the presence or absence of MERCs affect the dynamics of these second messengers and alter the energy landscape in these locations? And finally, how do different geometric features such as mitochondrial length and MERC surface area ratio affect Ca^2+^ handling and energy generation in spines? To answer these questions, we developed a spatiotemporal model of Ca^2+^ dynamics in a portion of a dendrite with spatially resolved ER and mitochondria in idealized geometries. We found that the MERC leads to a significant increase in mitochondrial calcium, and subsequently ATP production. Additionally, increasing the surface area of the MERC, increases the calcium influx into the mitochondria, providing insight into how the different extents of MERC can give rise to different calcium states. Finally, we predict metabolic parameters, such as cytosolic ATP hydrolysis, nucleotide transport from mitochondria to the cytoplasm, and rate of ATP synthesis to be key deciding factors in the delicate balance of calcium signaling and energy homeostasis in the postsynaptic spine.

## Results

We constructed a spatial model with five compartments: the postsynaptic density (PSD), the cytosol, one mitochondria, the ER, and a mitochondria ER Contact region (MERC) (Fig. 1a, b). In our simplified geometry (shown in Fig. 1c), the dendritic spine is modeled as a sphere attached to the large cylindrical dendritic shaft by a narrow cylindrical spine neck, geometrically defined in Supplementary Table 1. Although the morphology of a spine has been shown to govern the magnitude and stability of calcium transients in previous studies^26,33,39^, in this study we simplify the complex spine morphology to idealized geometries to focus on the role of mitochondria in neuronal calcium dynamics. While the interconnected ER tubules are widely distributed throughout the cytoplasm, we approximate the ER within the dendritic shaft as long cylinders, keeping ER to cytoplasmic volume ratios constant. The mitochondrion was modeled as a wide cylinder in the center of these four ER. Although dendritic mitochondria have a wide range of sizes from sub micron to to more than 10 μm with complex inner membrane and outer membrane morphologies^42^, we approximate a relatively small mitochondria of 0.6 μm localized at the base of a dendritic spine. The sizes of the compartments in the model are given in Supplementary Table 1 and were chosen based on experimental evidence^43–45^. The details of the model development and numerical methods are given in the supplementary material (Supplementary Tables 2 and 3). The initial conditions for the model were established by initializing the system with no stimulus for 120 s and identifying approximate steady states (Supplemental Fig. 1). Although this is not a true steady state, this approach allows us to mitigate some of the differences that arise due to changing compartment and boundary sizes without impacting computational cost of the system.

### Spatial dynamics of Ca^2+^ and IP_3_ in spines with and without MERC

We conducted simulations with a 1 Hz stimulus of glutamate for 5 s and ran the simulations until an approximate steady state is reached (Supplemental Fig. 1).

**Fig. 2:**
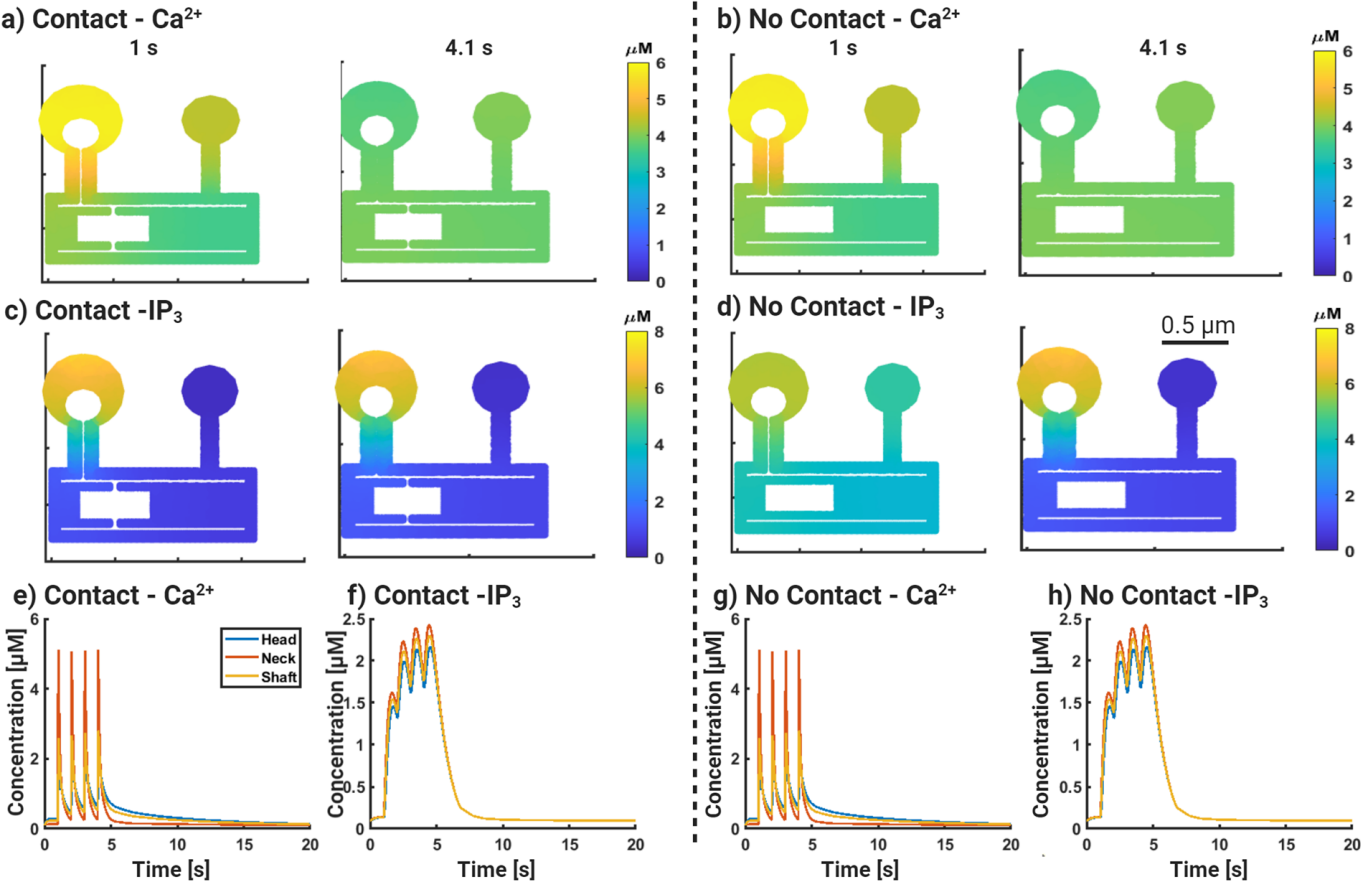
Receptor-based simulations of glutamatergic signaling in spatial calcium model. Finite element simulations of dendritic spines including mitochondria, ER, and mitochondrial-ER contacts(MERC). **a**) Ca^2+^ dynamics in the stimulated and neighboring spine in the presence of MERC. **b**) Same as (**a**) in the absence of MERC. **c**) IP_3_ dynamics in the stimulated and neighboring spine in the presence of MERC. **d**) Same as (**c**) in the absence of MERC. Time course of Ca^2+^ (**e**) and IP_3_ (**f**) with MERC. Time course of Ca^2+^ (**g**) and IP_3_ (**h**) without MERC.

In all simulations, only the larger spine with the spine apparatus was stimulated with the 1 Hz pulse train. We conducted these simulations in two geometries—one without the MERCs (no gray ring) and one with MERC consisting of 10% of the total surface area (Fig. 1c). Spatial maps at 1 s and 4.1 s were chosen to demonstrate the spatial dynamics of Ca^2+^ and IP_3_ (shown in Fig. 2). Corresponding temporal dynamics at different locations are also shown. We first looked at Ca^2+^ dynamics and IP_3_ distribution with and without MERC (Fig. 2).

The peak calcium concentration is higher in the spine head (5.5 μM) and about half that in the dendritic shaft (2.5 μM) proximal to the stimulated spine. In contrast, the neighboring spine has a peak Ca^2+^ concentration of about 4 μM with a distinctly lower gradient between spine head and shaft calcium.

When we look at the simulations without MERC (Fig. 2b), we note that the absence of MERC has negligible effects on the spine Ca^2+^ concentration dynamics in both the stimulated and neighboring spines.

Comparing the scenario with and without MERC, we note that the MERC compartment essentially allows for an increased and rapid early release of Ca^2+^ from the base of the stimulated spine (Fig. 2a, b) than in the case without MERC. When we look at the corresponding IP_3_ dynamics in the stimulated spine (Fig. 2c, d), we note that the immediate response of the stimulated spine, the spatial gradient of IP_3_, primarily from the spine head to the dendrite, consistent with the metabotropic glutamate receptor (mGluR) location and PIP_2_ hydrolysis from the plasma membrane. The neighboring spine receives IP_3_ only through diffusion and therefore has a lower concentration and no observable gradients of IP_3_. When there is no MERC, IP_3_ concentrations are similar to the case with MERC because IP_3_ is an early response to mGluR and not affected at these times by Ca^2+^.

### Presence of MERCs alter organelle Ca^2+^ dynamics and ATP production

Next, we investigated the effect of MERCs on Ca^2+^ dynamics in the ER and mitochondria in the same simulation condition as Fig. 2. In the dendrite, the ER acts as an intracellular calcium storage compartment and surface receptors on the ER, such as ryanodine and inositol 1,4,5- trisphosphate (IP_3_R), rapidly release calcium into the cytosol^46^. Sacroplasmic/endoplasmic reticulum Ca^2+^-ATPases (SERCAs) can result in high uptake of Ca^2+^ into the ER ( > 300 μM^47^) through the consumption of ATP. Ca^2+^ is then released from the ER through IP_3_R and ryanodine receptors (RyRs)^48^. Ca^2+^ regulation and mitochondrial ATP production are crucial for synaptic function and neuroplasticity^49^; mitochondria in synaptic terminals aid neurotransmission by producing ATP, buffering calcium, and local protein translation^10,50^. The metabolic cost of synaptic plasticity has been estimated to be between 3.4 and 9.4 × 10^5^ ATP molecules per min and the cost of postsynaptic current is 8.4 × 10^6^ ATP molecules per min (see Supplementary Table 1 in ref. ^51^. If we assume that the maximum metabolic cost is the sum of both these factors, then we obtain roughly 155,000 molecules of ATP/s as the energy requirement per spine. Therefore, in our model, in addition to tracking the concentrations of different species, we also calculate the ATP produced under different conditions and interpret them in the context of energy requirement for synaptic plasticity. We calculate the area under the curve (AUC) at 10s, 30s, and 60s for mitochondrial and cytosolic ATP to obtain the total number of molecules of ATP available in the system.

**Fig. 3:**
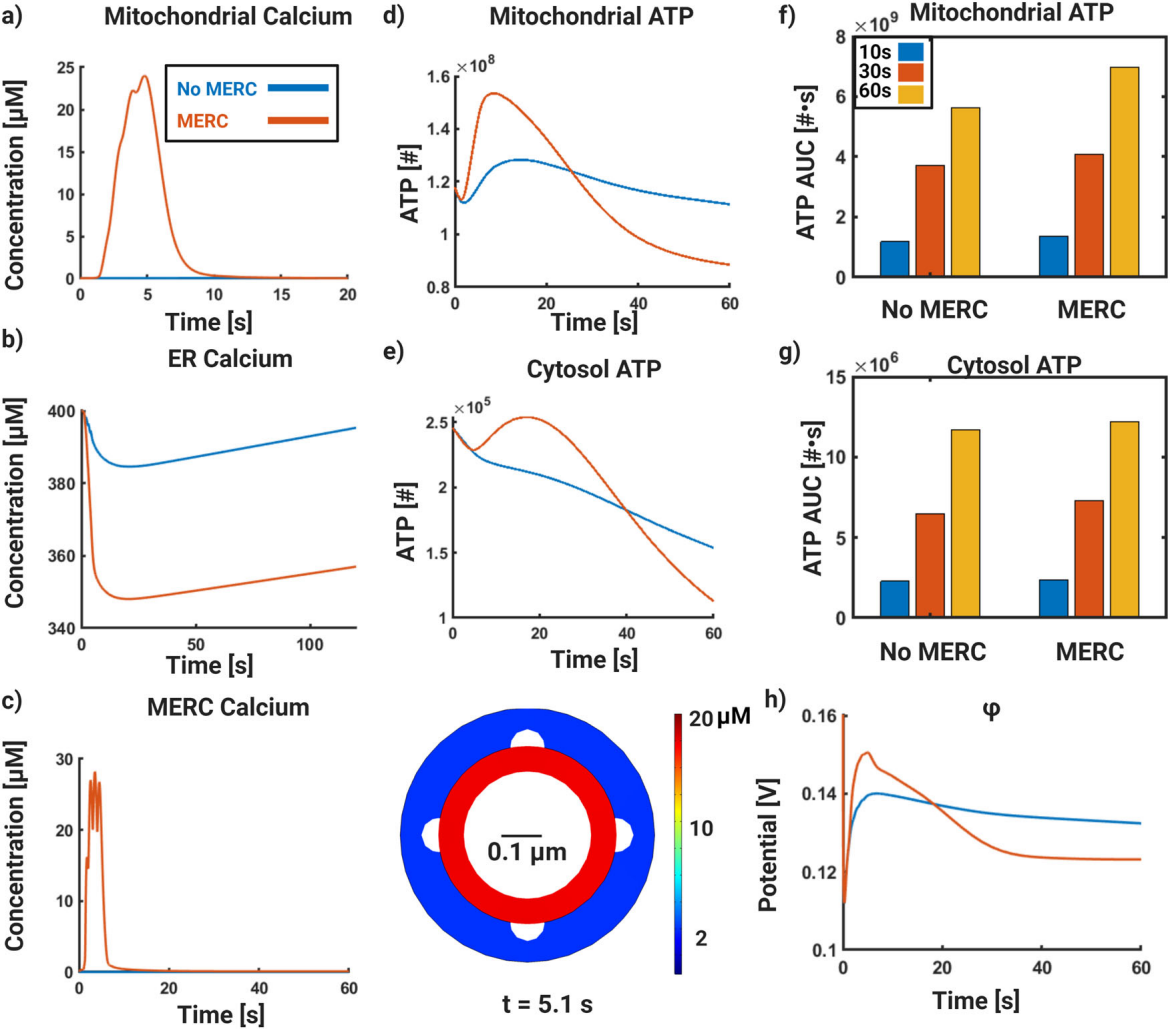
Receptor-based simulations of glutamatergic signaling in spatial calcium model. Finite element simulations of dendritic spines including mitochondria, ER, and mitochondrial-ER contacts(MERC). **a**) Mitochondrial Ca^2+^ dynamics in the presence of (red) and absence of (blue) MERC. **b**) ER Ca^2+^ dynamics in the presence of (red) and absence of (blue) MERC. **c**) Dynamics of MERC Ca^2+^ in the MERC case only (left); cross-section of MERC ring showing a higher concentration of Ca^2+^ in the MERC ring as opposed to the general cytoplasm (right). **d**) Mitochondrial ATP dynamics in the presence of (red) and absence of (blue) MERC. **e**) Cytosolic ATP dynamics in the presence of (red) and absence of (blue) MERC. **f**) AUC for mitochondrial ATP at 10 (blue), 30 (red), and 60 s (yellow) with and without MERC. **g**) AUC for cytosolic ATP at 10 (blue), 30 (red), and 60 s (yellow) with and without MERC. **h**) Mitochondrial membrane potential in the presence of (red) and absence of (blue) MERC.

In our simulations, when MERCs are present, we observed that the mitochondrial calcium is higher than in the absence of MERCs (Fig. 3a). This is the first indication from our simulations that spatial compartments such as MERCs can significantly alter Ca^2+^ dynamics in organelles, consistent with other hypotheses in the literature^19,52,53^. The ER Ca^2+^ also changes with more decline in the presence MERC (Fig. 3b). ER Ca^2+^ refills with different time scales for the two different cases reflecting the dynamics of MERC Ca^2+^. This is because the MERC is directly connected to the ER and there is significant calcium flux from the ER to the MERC. One of the most interesting feature of this model is that MERCs act as a Ca^2+^ microdomain by localizing high Ca^2+^ concentrations for a finite duration in a receptor dependent manner (Fig. 3c). Thus, our model predicts that the presence of MERC plays an important role in mitochondrial and MERC Ca^2+^ dynamics. As a result, we see an impact on mitochondrial and cytosolic ATP dynamics (Fig. 3d, e).

In the presence of MERC, the production of both mitochondrial and cytosolic ATP is rapid and has a higher peak. We note that the values of ATP in our model are consistent with estimates by Karbowski and others indicating that our model is operating in a physiologically relevant regime^51^. In addition to number of ATP molecules per time, we also show the time-integrated ATP values at 10, 30, and 60 s. At all times, the presence of MERC results in an increased AUC of ATP in both mitochondria and cytosol when the MERC is present. (Fig. 3f, g). The dynamics of the mitochondrial membrane potential (Ψ_*m*_) are also altered in the presence of MERCs (Fig. 3h), consistent with the mitochondrial ATP dynamics. The increased production and prolonged dynamics of ATP are weakly dependent on the calcium diffusion within the system. In Supplemental Fig. 2, we show that decreasing the diffusion coefficient by two orders of magnitude leads to a slight delay in cytosolic and mitochondrial ATP for dynamics (Supplemental Fig. 2a, b). We also note that compared to the Ca^2+^ dynamics in the ER and the MERC, the mitochondrial Ca^2+^ and ATP dynamics are smoother, indicating that the mitochondria retain the lower frequency information and not the higher frequency (Supplemental Fig. 2b, c)^54,55^. MERC calcium is not affected by the diffusion coefficient of calcium (Supplemental Fig. 2e).

Our model shows that the MERC compartment, which is technically part of the cytosol, is enriched in calcium even though there is no physical membrane separating the two compartments. By serving as a direct conduit for Ca^2+^ influx from the cytosol, we suggest that MERCs play an important role in localizing Ca^2+^ and thereby increasing ATP production locally at active synapses.

### Effect of mitochondrial size and MERC area fraction on Ca^2+^ and ATP dynamics

**Fig. 4:**
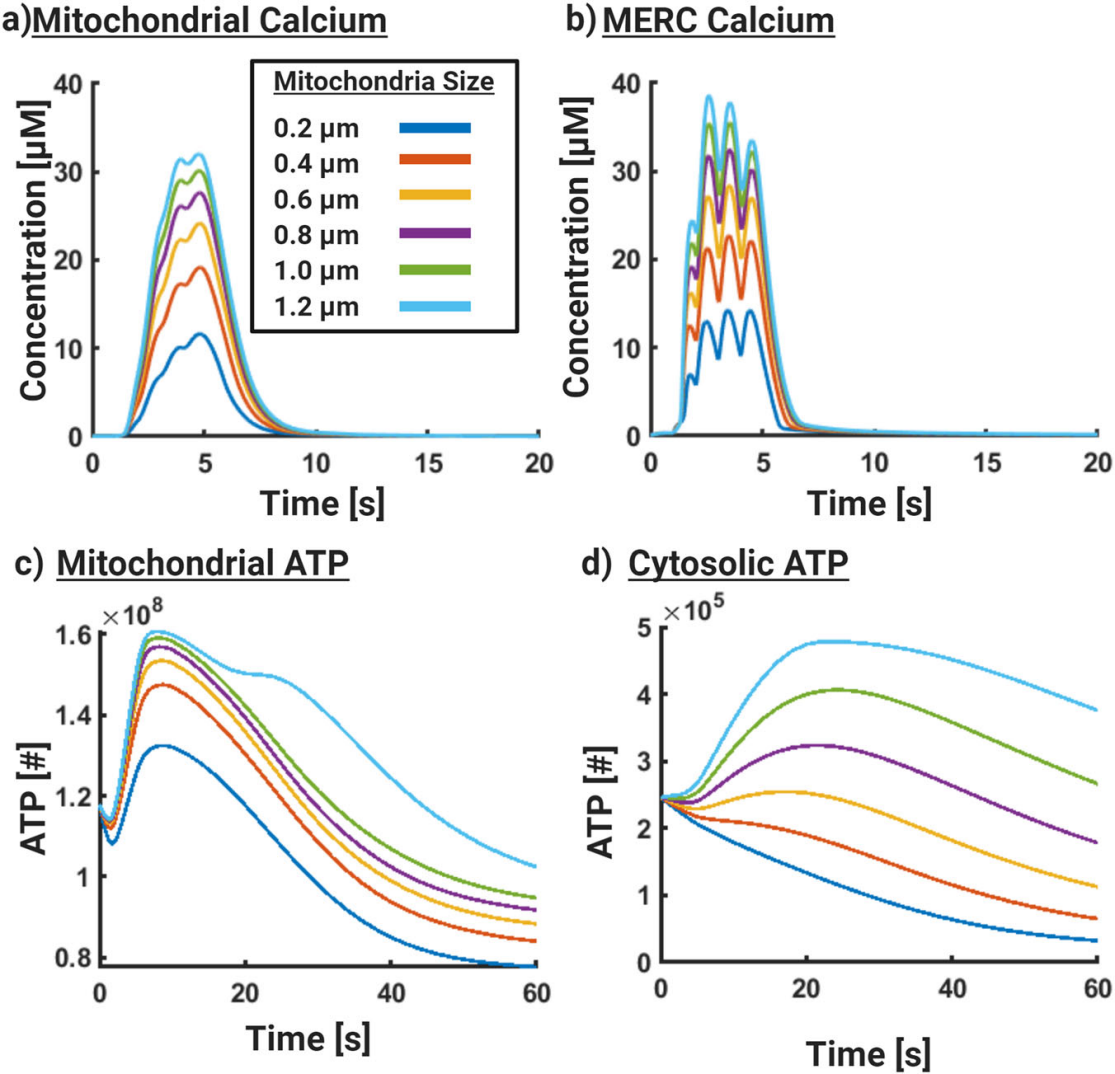
Mitochondrial length increases the magnitude of mitochondrial Ca^2+^ and ATP. In these simulations we increased the length of the mitochondria while keeping the MERC area fraction constant at 0.1. **a**) Volumetric average dynamics of mitochondrial Ca^2+^ increases with increasing mitochondrial length. **b**) Volumetric average dynamics of MERC Ca^2+^ with increasing mitochondrial length. **c**) Mitochondrial ATP, in number of molecules, increases with increasing mitochondrial length. **d**) Cytosolic ATP, in number of molecules, increases with increasing mitochondrial length.

**Fig. 5:**
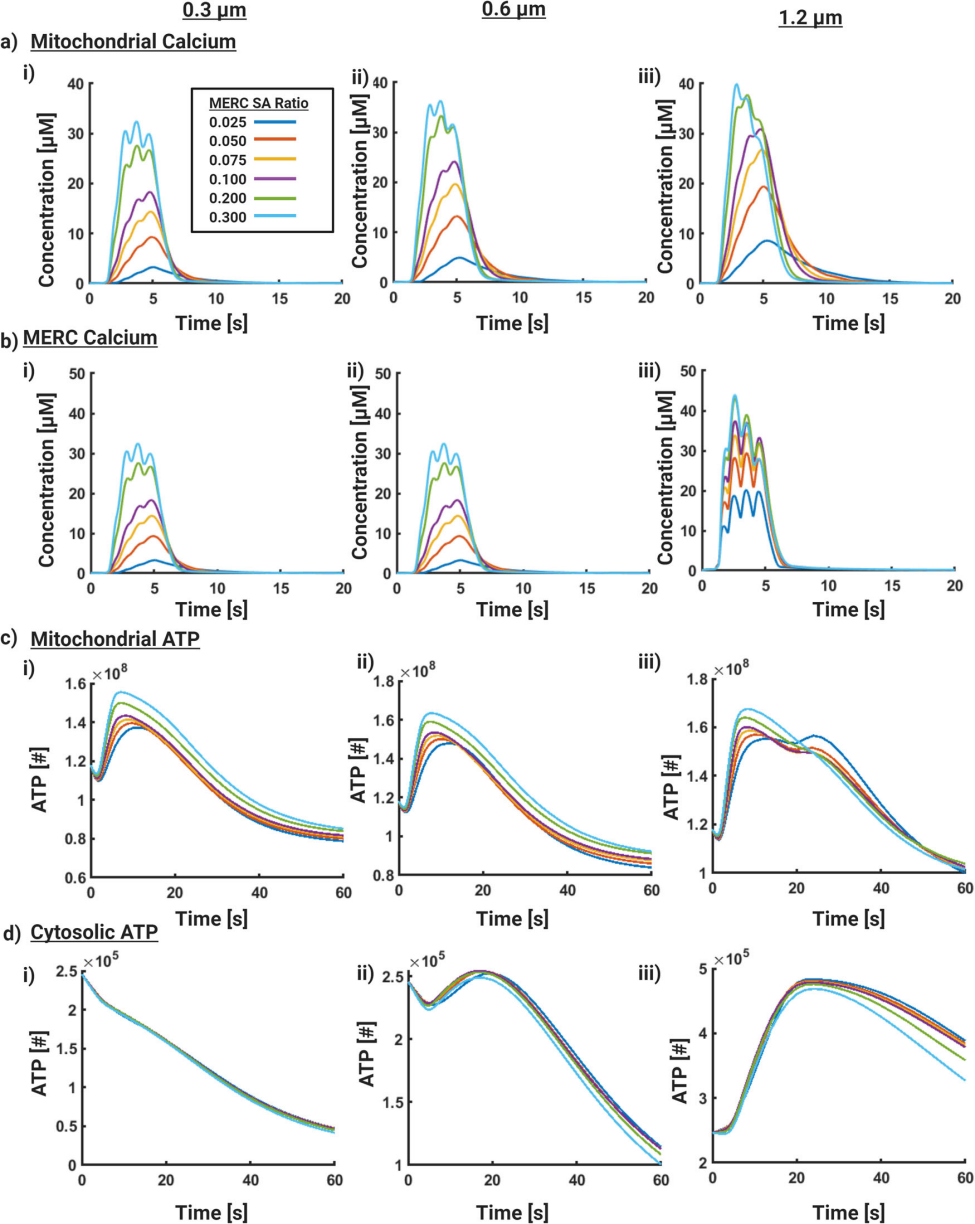
Effect of MERC area fraction on ATP production. Here we vary the size of the mitochondria and the area fraction of MERC systematically. Three different mitochondrial sizes (0.3, 0.6, and 1.2 μm) and six different MERC area fractions ranging from 0.025 to 0.3 were simulated. **a**) Mitochondrial Ca^2+^ dynamics, **b**) MERC Ca^2+^ dynamics, **c**) Mitochondrial ATP dynamics, and **d**) Cytosolic ATP dynamics for the different cases are shown.

The mitochondria present in dendrites of neurons are abundant and can vary in size^10,42^. The area fraction of MERCs (calculated as the ratio of MERC surface area in contact with mitochondria to the total mitochondrial surface area) is also known to vary^19^. Do these physical variables have an impact on the production of ATP in the mitochondria? To address this question, we conducted two sets of simulations: (i) vary mitochondrial length while maintaining MERC area fraction, and (ii) vary MERC area fraction in mitochondria of 3 sizes (Figs. 4 and 5 respectively).

We first explore the effect of mitochondrial size on organelle Ca^2+^ and ATP dynamics (Fig. 4). In these simulations, we maintained the MERC area fraction as 0.1 while varying the mitochondrial size from 0.2 to 1.2 μm. Our simulations showed that increasing mitochondrial size clearly favored increases in mitochondrial Ca^2+^ (Fig. 4a) and MERC Ca^2+^ (Fig. 4b). We note that the increase in the Ca^2+^ begins to saturate (see peak Ca^2+^ values in Fig. 4a) after a certain size of mitochondria suggesting that there is no additional advantage of large mitochondria in terms of Ca^2+^ concentration. This effect is also seen in the MERC Ca^2+^. Correspondingly, we observed increases in mitochondrial ATP production capacity but also a saturation at higher mitochondrial sizes (Fig. 4c). We note that for the larger mitochondria, a second, smaller peak appears at a later time point suggesting that mitochondrial size can alter the temporal dynamics. Thus far, our results are consistent with our expectation that increasing mitochondrial size increases the total amount of Ca^2+^ and ATP produced in the mitochondria. In this model, mitochondrial size is limited by our dendrite segment size, however, our results suggest that a larger mitochondria would produce more ATP.

When we look at the cytosolic ATP, we note that smaller mitochondria actually show a decrease in cytosolic ATP. As the mitochondrial size increases, cytosolic ATP increases and at larger mitochondrial sizes the kinetics are prolonged (Fig. 4d). This effect of mitochondrial size on cytosolic ATP can be understood as a result of the competition between the boundary fluxes on the mitochondrial membrane for adenine nucleotide translocator (ANT) and the hydrolysis rate occurring in the cytosol. As the mitochondrial size increases, the corresponding surface area increases, increasing the flux across the mitochondrial membrane into the cytosol. This change in flux also alters the kinetics of cytosolic ATP.

Thus, our model predicts that the presence of MERCs alone does not inherently confer an advantage for increased cytosolic ATP for all mitochondrial sizes. Rather, an equilibrium between rapid ATP production in the mitochondria through MERCs and a rapid ATP delivery to the cytosol through ANT (membrane boundary fluxes) are required to balance ATP production and supply in dendrites. ANT also drives ADP transport between the cytosol and mitochondria, which is a necessary substrate for ATP production. For effective ATP availability in the dendrites our model predicts two requirements—production of ATP in the mitochondria and availability of ATP in the cytosol. Production of ATP in the mitochondria depends on MERC area fraction and mitochondrial size. Availability of ATP in the cytosol depends on the flux of ATP from the mitochondria through the ANT and the consumption of ATP through a lumped hydrolysis rate.

We next investigated the role of MERC and mitochondrial sizes in governing ATP production capacity. Since MERCs provide a direct conduit to increase mitochondrial Ca^2+^, we varied the area fraction of MERCs with mitochondria of different sizes (0.3, 0.6, and 1.2 μm). These lengths are supported by observations that Ca-CaMKII activity can decrease mitochondrial length over a range of synaptic activity levels^56^. Particularly, we sought to find if increasing MERC area fraction could confer an advantage to smaller mitochondria, compensating for volumetric disadvantage with increased calcium. Experimental evidence indicates that MERCs range from 2 to 20% of mitochondria surface area depending on the metabolic state and type of cell^45,52,57^. We conducted simulations in small (0.3 μm), medium (0.6 μm), and large mitochondria (1.2 μm), with a range of MERC area fractions ranging from 2.5 to 30% (Fig. 5) for a fixed value of the ATP consumption rate of 200 μM/s. We note that, while area fraction ranges have been experimentally quantified, they need not necessarily form a ring-like structure that we model here^19,45,58,59^. As shown in Supplemental Fig. 5, cytosolic Ca^2+^ and IP_3_ are not dramatically affected by overall size of MERCs.

We observe that increasing the MERC area fraction increases mitochondrial Ca^2+^ across all sizes and the peak concentration of Ca^2+^ increases with increasing mitochondrial size (Fig. 5a). MERC Ca^2+^ dynamics also show an increase with increasing MERC area fraction for all mitochondrial sizes with a proportionally small increase in the peak Ca^2+^ (Fig. 5b). Mitochondrial ATP production increases with the MERC area fraction within a given mitochondrial size to a smaller extent and also increases with larger mitochondrial sizes (Fig. 5c). Interestingly, the dynamics of mitochondrial ATP show a faster decay with increasing MERC area fraction for small and medium mitochondria (Fig. 5c, i,ii). For the large mitochondria, we observe a second and prolonged peak of mitochondrial ATP as the area fraction of MERC increases (Fig. 5c,iii). Thus, the combination of MERC area fraction and mitochondrial sizes results in synergistic effects on mitochondrial ATP production. However, looking at the cytosolic ATP dynamics, we note that not all mitochondrial sizes or MERC fractions are conducive to increased ATP availability (Fig. 5d). Small mitochondria show practically no increase in cytosolic ATP and are unaffected by MERC area fraction (Fig. 5d, i). Medium size mitochondria begin to show some response in that increasing the MERC area fraction prolongs the decay time but no dramatic increase in cytosolic ATP is observed (Fig. 5d,ii) nor is a dependence on MERC SA fraction. Large mitochondria show an increase in cytosolic ATP even for small MERC area fractions but increasing MERC area fraction in large mitochondria does not later ATP concentration in the cytosol dramatically (Fig. 5d, iii).

### Combination of increased mitochondrial size and MERC area fraction buffers ATP production capacity

**Fig. 6:**
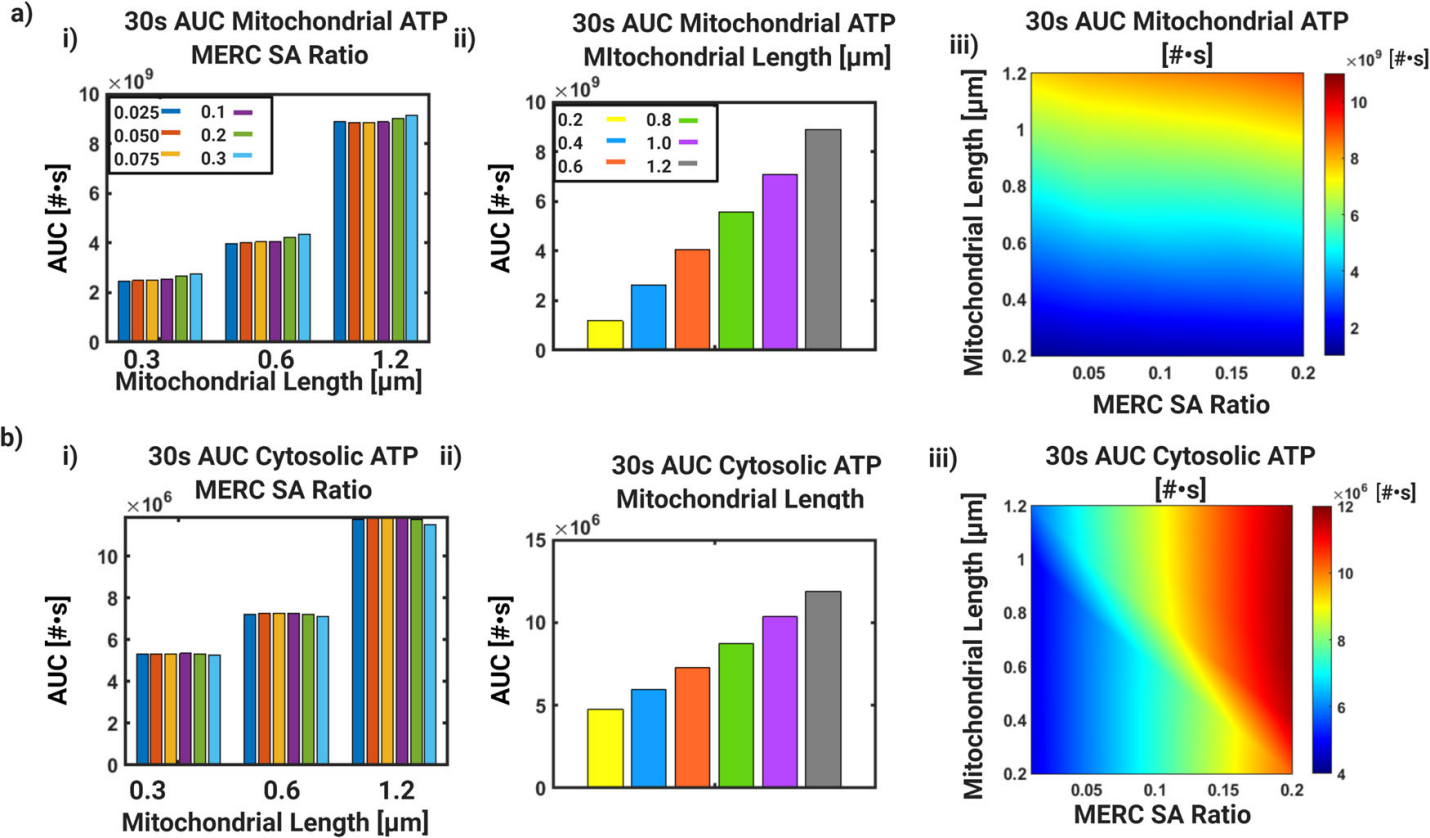
Area Under the Curve (AUC) for varying mitochondrial length and MERC SA. For variations in both Figs. 4 and 5, we quantify the total metabolic output through the computation of AUC in terms of molecules of ATP for 30 s for the respective compartment volumes. **a**) Mitochondrial ATP AUC quantified for a window of 30s starting from time of stimulus. **i**) For 3 mitochondrial sizes, 0.3, 0.6, and 1.2 μm, the AUC for increasing surface area ratios, 0.01, 0.05, 0.1, 0.2, 0.3 as shown in Fig. 5. **ii**) The AUC for a fixed SA ratio of 0.1 and increasing values of mitochondrial length, as shown in Fig. 4. **iii**) Phase diagram of AUC shows combined effect of MERC SA ratio and mitochondrial length on mitochondrial ATP AUC at 30 s. For both Figs. 4 and 5, we quantify the total metabolic output through the computation of AUC in terms of molecules of ATP for 30 s for the respective compartment volumes. **b**) Cytosolic ATP AUC quantified for a window of 30s starting from time of stimulus. **i**) For 3 mitochondrial sizes, 0.3, 0.6, and 1.2 μm, the AUC for increasing surface area ratios, 0.01, 0.05, 0.1, 0.2, 0.3, as shown in Fig. 5. **ii**) The AUC for a fixed SA ratio of 0.1 and increasing values of mitochondria, as shown in Fig. 4. **iii**) Phase diagram of AUC shows combined effect of MERC SA ratio and mitochondrial length on cytosolic ATP AUC at 30 s).

For the different combinations of mitochondrial length and MERC SA ratio, we evaluated the AUC for mitochondrial and cytoslic ATP at 30 s (Fig. 6). For mitochondrial ATP, the effect of increase SA ratio was most dramatic for the large mitochondria, consistent with Fig. 5d when compared to the small and medium size mitochondria (Fig. 6a,i). For a fixed SA ratio of 0.1, when we look at mitochondrial ATP AUC for increasing mitochondrial length, we note that the effect of mitochondrial length are much stronger (Fig. 6a,ii). Finally, when we look at the combined effects of varying both SA ratio and mitochondrial length, it is evident that for mitochondrial ATP production, larger mitochondria are favorable across the range of MERC SA ratio for increased ATP (Fig. 6a,iii) and the reverse is true for smaller mitochondria. Increase in MERC SA ratio doesn’t necessarily rescue the small mitochondria’s ATP production capability. However, at intermediate mitochondrial lengths, increasing MERC SA ratio increases mitochondrial ATP production, suggesting some synergistic benefit (Fig. 6a,iii).

When we consider cytosolic ATP, we observe that for a given mitochondrial length, varying MERC SA ratio did not make a difference for ATP availability (Fig. 6b,i) but increasing mitochondrial length for a fixed MERC SA ratio of 0.1 showed that cytosolic ATP increases with mitochondrial length (Fig. 6b, ii). The combined effects of varying mitochondrial length and MERC SA ratio shows a completely different phase map for cytosolic ATP when compared to mitochondrial ATP (compare (Fig. 6a,iii) to (Fig. 6b, iii)). For cytosolic ATP, high MERC SA ratio provided benefits of increasing ATP availability even for small mitochondria whereas small MERC SA ratio didn’t offer much benefit even for large mitochondrial length. This is consistent with our previous observation of the role played by boundary conditions. As we increase mitochondrial length, the range of MERC SA ratios for which high cytosolic ATP is achieved widens.

### Energy availability at the synapse is a combination of mitochondrial geometry, MERC area fraction, and kinetics of ATP production and consumption

**Fig. 7:**
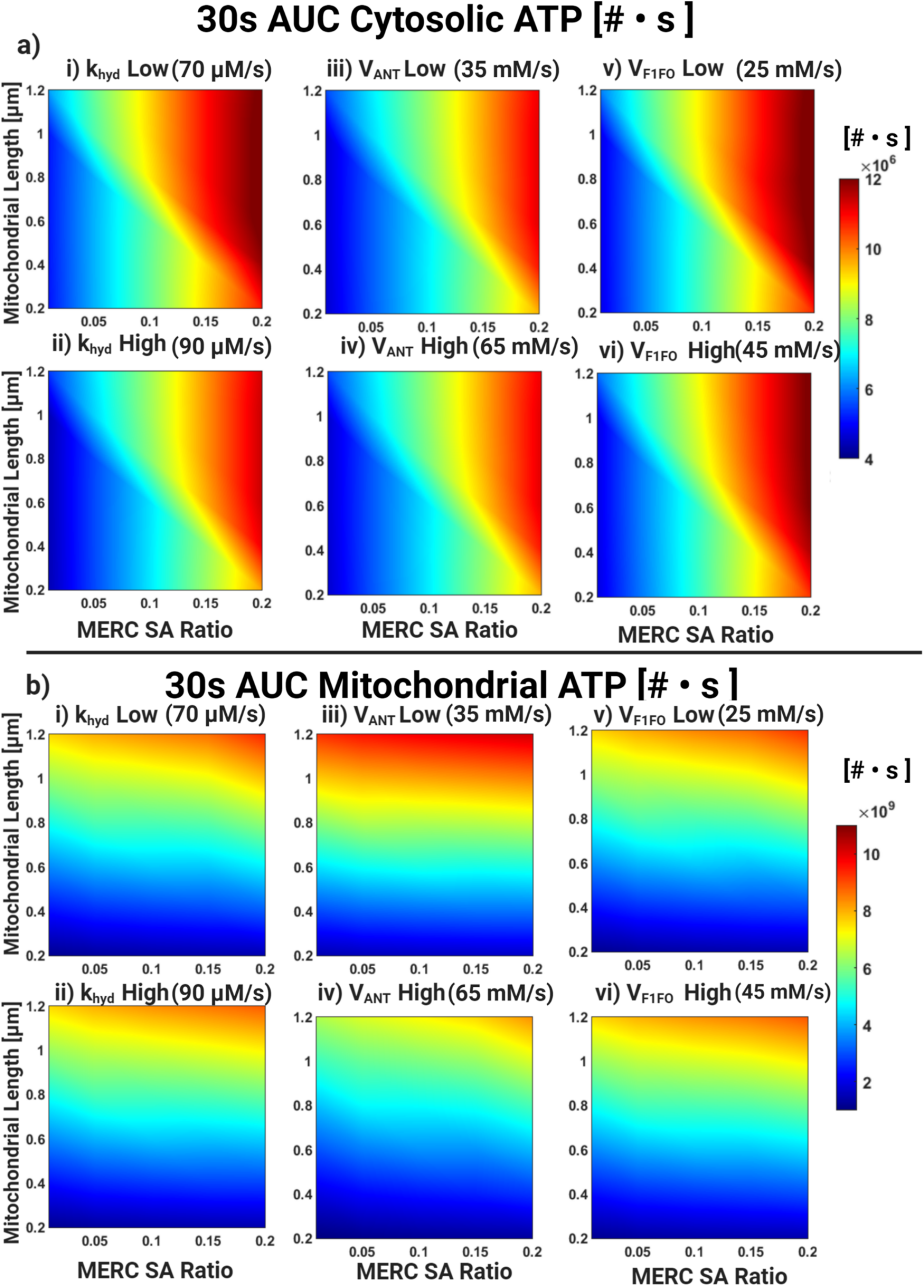
Phase diagram for AUC of ATP for 30 s post-stimulus with varying metabolic parameters. **a** Cytosolic ATP AUC phase map for **i**) *k*_*HYD*_ = 70 *μM*/*s*, **ii**) *k*_*HYD*_ = 90 μM/s, **iii**) *V*_*ANT*_ = 35*mM*/*s*, **iv**) *V*_*ANT*_ = 65*mM*/*s*, **v**) *V*_*F*1*FO*_ = 25*mM*/*s*, **vi**) *V*_*F*1*FO*_ = 45*mM*/*s*. **b**) Same as (**a**) for mitochondrial ATP.

Finally, we asked the following question: how is the balance between the kinetics of ATP production through F1FO, ATP transport through ANT, and ATP consumption (modeled using a lumped hydrolysis term, *k*_*HYD*_) influenced by mitochondrial size and MERC area fraction? We conducted a series of simulations to map the corresponding phase spaces that hold the answer to this question. For all simulations, we varied mitochondrial size from 0.3 to 1.2 μm and MERC area fraction from 0 (no contact) to 0.2. Then, we calculated the AUC for mitochondrial ATP and the AUC for cytosolic ATP as our scalar readout at 10s (Supplemental Fig. 3), 30 s (Fig. 7), and at 60 s (Supplemental Fig. 4). We observe that for the different values of *k*_*HYD*_, *V*_*ANT*_, and *V*_*F*1*FO*_, the nature of the phase map with respect to mitochondrial length and MERC SA ratio remains qualitatively unaltered but shows quantitative changes.

We first explore the role of the rate of ATP consumption in our system, by varying *k*_*HYD*_ (Supplementary Table 4) in the cytosol. This lumped parameter effectively captures the energy consumption in the postsynaptic spine; over 60% of neuronal energy consumption is consumed during active calcium signaling, which occurs at the Plasma Membrane (PM) and ER boundaries^60^. The different sources of energy consumption in a spine are reviewed in refs. ^51,61,62^. Estimations of this rate are difficult to obtain directly, but using estimates in ref. ^51^, we calculated an estimate of the energy consumption of a potentiated spine. The estimate of total energy consumption in a single spine is given as 30,000 mol/min^51^. For an average spine volume of 0.1 m^3^, this is approximately 83 M/s of ATP consumption in the spine. The rate that we have used in this model is 80 M/s, which is in agreement with the above estimate. We varied the value of *k*_*HYD*_ by 10 μM/s in either direction with respect to our control value of 80 μM/s. We note that for low *k*_*HYD*_ for different mitochondrial lengths and MERC SA ratio (Fig. 6a i) the blue region is narrower for when compared to high *k*_*HYD*_ (Fig. 7a ii). This effect is also seen for mitochondrial AUC (Fig. 7b i, ii) and can be understood as low *k*_*HYD*_ means more ATP is available since the consumption rate is low.

Next, we changed the value of *V*_*ANT*_ to study the role of ATP transport across the mitochondrial membrane on the energetic balance between mitochondria and cytosol. We varied the value of *V*_*ANT*_ 15 μM/s in either direction with respect to our control value of 50 μM/s. V_*ANT*_ has a limited influence on the total production of ATP in the system. However, increasing *V*_*ANT*_ slightly widens the range of mitochondrial length and SA ratio for which one can obtain high ATP AUC (Fig. 7a iii, iv) but dramatically reduces mitochondrial ATP AUC (Fig. 7b iii, iv). This is because increasing *V*_*ANT*_ increases the flux of ATP from the mitochondria to the cytosol. Thus, *V*_*ANT*_ is a critical parameter in transporting the ATP generated in the mitochondria to the cytosol altering the net energy availability.

Finally, to investigate the role of the rate of ATP production by the F1,FO ATP synthase, we varied the kinetic parameter *V*_*F*1,*FO*_ (Fig. 7a v, vi, Supplementary Tables 2 and 4). This variation effectively captures the changes to either the activity of the ATP synthase or the number of ATP synthases. We varied the value of *V*_*F*1*FO*_ by 10 μM/s in either direction with respect to our control value of 35 μM. Cytosolic ATP AUC showed small changes to mitochondrial length and MERC SA ratio for low and high values of *V*_*F*1*FO*_ (Fig. 7a, v,vi) and so did mitochondrial ATP AUC (Fig. 7b, v,vi).

Thus, our model shows that the metabolic parameters associated with ATP production, transport, and consumption in conjunction with MERC surface area ratio and mitochondrial length are important for ATP availability.

## Discussion

**Fig. 8:**
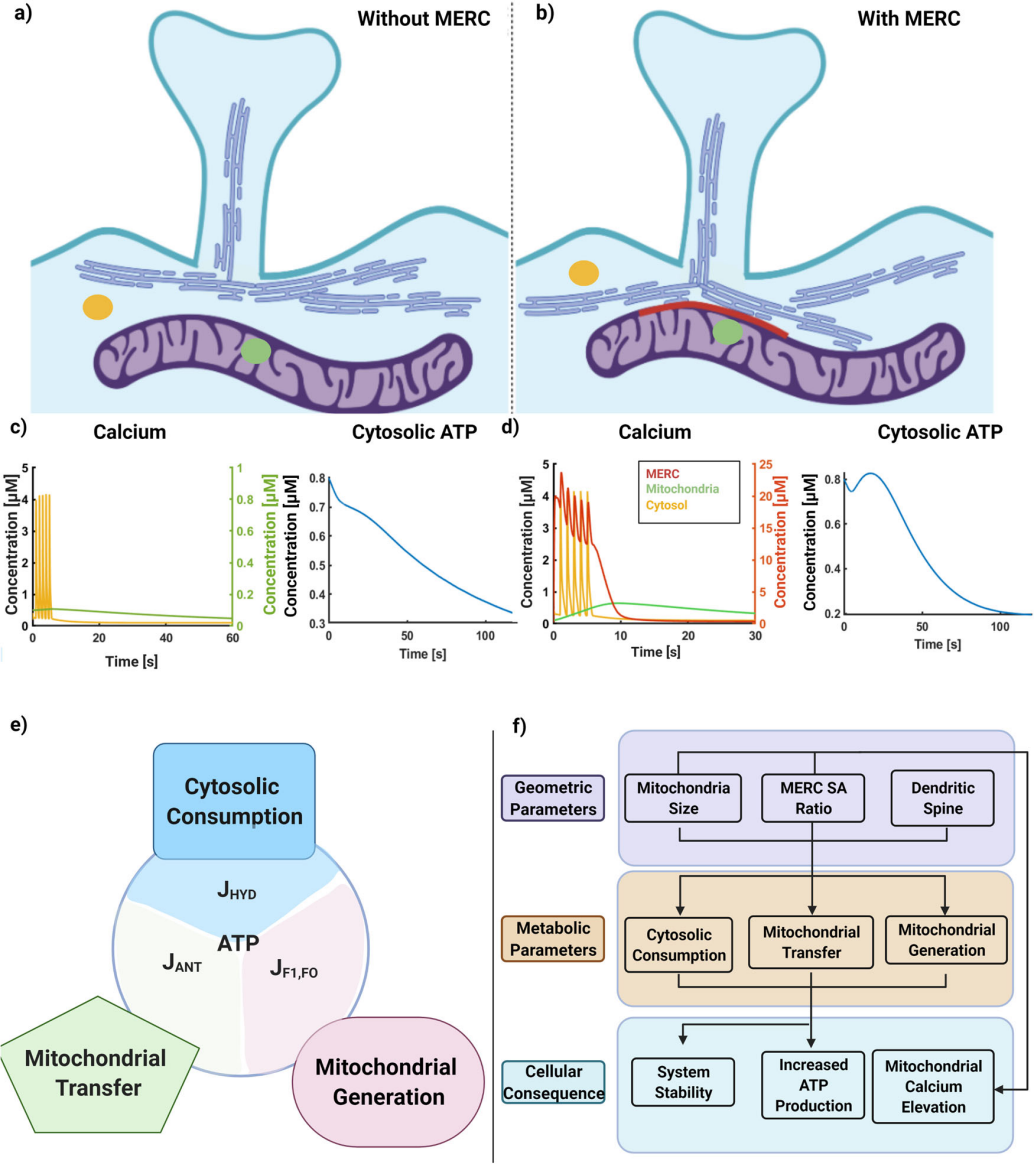
Impact of MERCs and geometric regulation of mitochondrial ATP production. **a**) Schematic for dendrite with ER not forming contacts with mitochondria. **b**) Schematic for dendrite with ER forming contacts with mitochondria, highlighted in red. **c**) **i**) Calcium profiles for cytosol (in yellow) and mitochondria (in green) for the system without any contact sites. **ii**) ATP profiles in the cytosol for the system without any contact sites. **d**) **i**) Calcium profiles for cytosol (in yellow), mitochondria (in green), and MERC (in red) for system with contact sites. **e**) Schematic outlining key fluxes that balance ATP in the mitochondria and cytosol explored in Fig. 7. **f**) Flow diagram describing the interconnected influence of cell and organelle geometry on ATP production with added feedback through calcium signaling. Figure created using biorender.com.

In this study, we investigated how the presence of MERCs can alter Ca^2+^ and ATP dynamics in the postsynaptic spine and surrounding areas using computational modeling. Our model predicts inter-organellar coupling between ER and mitochondria through the presence of MERCs plays an important role in modulating the postsynaptic energy landscape. The presence of MERCs leads to increased mitochondrial Ca^2+^ transients and increased mitochondrial ATP production, consistent with experimental observations^56,58,63^. We also showed that mitochondrial size and MERC area fraction play a critical role in the balance of production of ATP in the mitochondria and availability of ATP in the cytosol by regulating the fluxes into and outside of the mitochondria^56^. And finally, we predict that metabolic parameters associated with mitochondrial function are also important determinants of the postsynaptic energy landscape (Fig. 8).

Recently, the mitochondrial dynamics, including fission^56^, ROS generation^64^, cytochrome c release^65^, and MERCs^58^ have been implicated in postsynaptic response to different stimuli, particularly in the context of energy requirements of synaptic transmission. Furthermore, the presence of MERCs has been noted as a critical factor in the mitochondrial response and loss of MERCs shows a reduced mitochondrial Ca^2+^ response in neurons^58^. Dendritic mitochondria can range in size from 1 μm to over 10 μm in length (median 2.5 μm)^11,42,56^. While our spatial model does not cover the entire dendrite (we chose a 1.5 μm long dendrite segment for computational ease), we note that our model predicts that dendritic energy availability is directly proportional to mitochondrial length. Divakaruni et al. report that mitochondrial length is linearly proportional to dendritic length^56^; thus the scaling we find here suggests a common size principle for dendritic energy availability. Separately, Hirayabashi and colleagues reported that PDZ Domain-Containing Protein 8 (PDZD8) is required for MERC formation and that the MERC area fraction ranges from 0.02 to 0.16 in HeLa cells, and 0.04 in neurons^58^.

Perturbation of MERCs halts ER-mitochondria Ca^2+^ transfer and impairs activity-dependent cytoplasmic Ca^2+^ dynamics in neurons^15^. In addition to having multiple contact sites, the ER forms an elaborate network around mitochondria in neuronal processes^66^. MERC distribution and ER topology in dendrites are also implicated in the regulation of mitochondrial dynamics and the formation of a spatially stable mitochondrial pool near active dendritic spines to fuel synaptic plasticity^67^. We note that increasing MERC-to-mitochondria surface area ratios influences ATP production dynamics as well as total ATP level. Although MERCs can have a substantial impact on mitochondrial ATP generation, in contrast to cases without MERC, the MERC area fraction does not show a linear dependence on ATP production as was observed for mitochondrial size.

Neuronal activity, while consuming ATP, also stimulates ATP production via a Ca^2+^ dependent increase in oxidative phosphorylation^10,68^. We also show that biochemical parameters associated with neuronal metabolism such as ATP production, nucleotide transport, and ATP hydrolysis also play an important role in the cytosolic energy landscape by allowing for a graded behavior. As demonstrated in our model, the spatiotemporal compartmentalization of Ca^2+^ flux and ATP source via MERCs provides a unique balance between local energy metabolism and synaptic activity. This balance is further regulated in cells by Ca^2+^-dependent mitochondrial fission to complete the feedback loop of signaling and mitochondrial size regulation, and will require a deeper understanding of the underlying coupled mechanochemical processes^69^. The subprocesses of synaptic plasticity, including actin remodeling, protein synthesis, receptor trafficking, and phosphorylation require a surge of ATP production^61,62^. In our work, MERC’s are able to transiently raise ATP generation at the cost of lower overall energy production.

We note that our model has made some simplifying assumptions to keep the simulations tractable. First, all our geometries are simplified, allowing us to construct a spatial model of multiple organelles. We note that current efforts include using realistic geometries of spines and organelles derived from microscopy to investigate how these play a role in Ca^2+^ and ATP dynamics^70–72^. Second, we note that we assume that Ca^2+^ and ATP are present in large enough amounts to justify a deterministic approach. We recognize that a stochastic approach would likely give rise to insights in limit cases of few molecules or in noisy environments^39,40^. Finally, we note that a more complete Ca^2+^ influx model would account for voltage-gated calcium channel dynamics as well^24,26,73,74^. This is a focus of technical development and ongoing effort in our group.

This work sets the stage for future investigation on the coupling between biochemical signaling^75^, biophysical mechanisms of organelle transport and tethering, and metabolic pathways, giving a glimpse into the complex regulation of synaptic plasticity by functional inter-organellar coupling.

## Methods

In this section, we discuss the details of the model development, assumptions, parameters, and numerical methods.

### Geometry development

We built a model with five compartments: the postsynaptic density (PSD), the cytosol, one mitochondria, the ER, and a mitochondria ER Contact region (MERC) (Fig. 1). In our simplified geometry, the dendritic spine is modeled as a sphere attached to the dendritic shaft by the spine neck. The spine neck and the dendritic shaft were modeled as cylinders (Supplementary Table 1).

Although the morphology of a spine has been shown to govern the magnitude and stability of calcium transients in dendrites in previous studies^26,33^, in this study we simplify complex spine morphology in idealized geometries to focus on the role of mitochondria in neuronal calcium dynamics. While the ER is distributed throughout the cytoplasm, we approximate the ER within the dendritic shaft as long cylinders, keeping volumetric ER to cytoplasm ratios constant. The sizes of these compartments are given in Supplementary Table 1. This model assumes an active, potentiated spine and based upon information found in Toresson et al., Cooney et al., and Mahajan et al. a potentiated spine often does contain spine apparatus and mitochondria by the base of the dendritic shaft^13,14,24^. While we acknowledge that these simplifications do not capture geometric complexity of a dendrite^45^, they allow us to explore the role of spatial organization of the spine in a computational framework.

### Neuronal stimulus patterns

While there are a multitude of signaling frequencies and patterns, in this work we focus on a simple periodic stimulus, 1 Hz for 5 s^76^ to emulate signaling in an established spine. Mathematically, we approximate glutamate stimulus as discrete pulses in a well-mixed system within COMSOL. Since we are not modeling synaptic vesicles or presynaptic neuron signaling, we apply a series of delta functions temporally spaced according to the desired pulse train (i.e. 1 Hz stimulus has delta functions spaced 1 seconds apart). Glutamate then decays with a time constant of 1.2 ms^77^. Key contributing factors to this decay are rapid reabsorption by the signaling neurons and astrocytic cells as well as diffusion^78^.

### Calcium dynamics

After glutamate binds to receptors on the post-synaptic density (PSD), there is a large calcium influx from the extracellular space and endoplasmic reticulum. This signaling initiates at the dendritic spine and propagates to the dendritic shaft, where the mitochondria are primarily located. We assume that the extracellular calcium does not deplete and does not alter the flux of synaptic calcium signaling. Extracellular calcium concentrations are typically several orders of magnitude higher than intracellular calcium^79^. The calcium dynamics in our model are based on work done by refs. ^27,29,80^, and parameters were adapted and suitably modified to reflect neuronal calcium metabolism.

- **Postsynaptic area and Receptor Dynamics:** This compartment includes membrane-bound molecules on the postsynaptic area: NMDA receptor (NMDAR), free mGluRs (R_2_), glutamate-bound mGluRs (DIM), phosphorylated mGluRs (DIM_*p*_), and second messenger Diacylglycerol (DAG). These molecules localize to the PSD area of spine heads. Calcium enters the spine head through NMDAR. The NMDAR dynamics are described in a multistate model^81^. NMDAR-induced calcium influx is modeled as:
  1 $${J}_{NMDA}={I}_{NMDA}$$
  where, *I*_*NMDA*_ is the current associated with calcium influx. Mathematical descriptions of all terms are expanded in Supplementary Table 2. Glutamate binds to the mGluR on the spine head, which induces G_*qα*_ to separate from G_*qβγ*_ and activate PLC_*β*_ enzyme. PLC_*β*_ enzyme cleaves PIP_2_ (a membrane phospholipid) and generates DAG and IP_3_. While DAG remains on the membrane and activates Protein kinase C (PKC), which then may phosphorylate mGluR; phosphatases dephosphorylate mGluR. This phosphorylation can inhibit the dimerization of mGluR. IP_3_ diffuses through the cytosol and initiates Ca^2+^ release from internal stores by binding to receptors (IP_3_R) on the ER membrane. IP_3_ in the cytosol is degraded to IP_2_ and IP_4_. The degradation is assumed to recycle phospholipids such that IP_3_ production is not rate limited by PIP_2_ availability. Ca^2+^ can also be released from ER by Ryanodine receptors and conversely can be actively transported back into the ER by Sarcoplasmic/endoplasmic reticulum calcium-ATPase (SERCA) on the ER membrane. Equations associated with the mGluR cascade are included in Supplementary Table 2. The calcium flux is modeled as a boundary condition on the endoplasmic reticulum membrane, in contrast to the NMDAR on the PSD.
- **Cytosolic Calcium Dynamics:** Ca^2+^ transients that accumulate due to influx at the PSD are able to diffuse throughout the cytosol. While we do not explicitly model calcium buffers, buffering capacity is included through boundary conditions between compartments. Cytosolic Ca^2+^ dynamics in 3D is defined as:
  2 $$\frac{\partial [C{a}_{c}^{2+}]}{\partial t}={D}_{C{a}^{2+}}{\nabla }^{2}[C{a}_{c}^{2+}]-{J}_{Buff}$$
  where ${D}_{C{a}^{2+}}$ is diffusion coefficient and ∇^2^ is Laplacian operator in 3D. The boundary condition for Ca^2+^ influx through NMDAR in postsynaptic area and Ca^2+^ efflux from cytosol is defined as,
  3 $${D}_{C{a}^{2+}}({\bf{n}}.\nabla [C{a}_{c}^{2+}]){| }_{PSD}=({J}_{NMDA}-{J}_{eff}){m}_{p}$$
  where *m*_*p*_ is a geometric factor for fluxes on the boundary and is defined as the ratio of the cytosol volume to the postsynaptic surface area. J_*NMDA*_ is defined as the flux as a result of the NMDA receptor activity. J_*eff*_ is defined as the activity as a result of plasma membrane calcium ATPase. The boundary fluxes on ER for cytosolic Ca^2+^ are defined as:
  4 $${D}_{C{a}^{2+}}({\bf{n}}.\nabla [C{a}_{c}^{2+}]){| }_{ER}={b}_{c}(\alpha {J}_{I{P}_{3}}+\alpha {J}_{Ryn}-{J}_{SERCA}){m}_{e}$$
  where b_*c*_ is the buffering capacity of cytosol and *α* is ER to cytosol volume ratio. ${J}_{I{P}_{3}R}$ is the Ca^2+^ flux from the ER into the cytosol through the IP_3_ receptor, *J*_*RYR*_ is the Ca^2+^ flux from the ER into the cytosol through the Ryanodine receptor, *J*_*SERCA*_ is the Ca^2+^ flux from the cytosol into the ER through the SERCA ATPase pumps, and *m*_*e*_ is a geometric factor for fluxes on the boundary and is defined as the ratio of the cytosolic volume to the ER surface area. ${J}_{I{P}_{3}R}$ and *J*_*RYR*_ are defined with respect to ER volume and *J*_*SERCA*_ is defined with respect to the cytosol volume. The mitochondrial boundary fluxes for cytosolic Ca^2+^ are defined as:
  5 $${D}_{C{a}^{2+}}({\bf{n}}.\nabla [C{a}_{c}^{2+}]){| }_{Mit}={b}_{c}(-\beta {J}_{MCU}+\beta {J}_{NCX}-\beta {J}_{mPTP})$$
  in which *β* is mitochondria to cytosol volume ratio. *J*_*MCU*_ is Ca^2+^ flux from the cytosol into the mitochondrion through the MCU channel, *J*_*NCX*_ is Ca^2+^ flux from the mitochondrion into the cytosol through Na^+^/Ca^2+^ exchanger, and *J*_*mPTP*_ is Ca^2+^ bidirectional flux of the mitochondrial Permeability Transition Pore. All of the fluxes within Eq. 5 are defined with respect to mitochondrion volume.
- **Calcium buffering:** Calcium buffering is explicitly included as a volumetric reaction between free calcium and buffer terms, denoted as J_*Buff*_.
  6 $$\frac{\partial B}{\partial t}={D}_{B}{\nabla }^{2}B-{J}_{Buff}$$
  Implicitly, we consider buffering terms within the boundary conditions between compartments. These buffering terms are denoted as *b*_*m*_, *b*_*c*_, *b*_*er*_ and b_*MERC*_.
- **Mitochondrial Calcium Dynamics:** The spatiotemporal dynamics of mitochondrial Ca^2+^ is given by
  7 $$\frac{\partial [C{a}_{m}^{2+}]}{\partial t}={D}_{C{a}^{2+}}{\nabla }^{2}[C{a}_{m}^{2+}]$$
  The boundary fluxes on mitochondrion surface are defined as:
  8 $${D}_{C{a}^{2+}}({\bf{n}}.\nabla [C{a}_{m}^{2+}]){| }_{Cyt}={b}_{m}({J}_{MCU}-{J}_{NCX}+{J}_{mPTP})$$
  in which b_*m*_ is the buffering capacity of Ca^2+^ in the mitochondrion. We do not consider the distinction between the outer and inner mitochondrial membrane. Voltage-dependent anion channels transport calcium highly efficiently during neuronal activation. Thus, we may assume, as done in refs. ^27,28^, that the calcium concentrations in the cytosol and outer mitochondrial calcium approach a rapid equilibrium. The inner mitochondrial membrane calcium fluxes are dependent solely on the cytosolic calcium. The same applies to fluxes regarding the mitochondria ER contact microdomain, which is represented in this model as a separate compartment.
- **ER Calcium Dynamics:** The only variable in ER compartment is Ca${}_{e}^{2+}$. Ca^2+^ in the ER is defined as:
  9 $$\frac{\partial [C{a}_{e}^{2+}]}{\partial t}={D}_{C{a}^{2+}}{\nabla }^{2}[C{a}_{e}^{2+}]$$
  The boundary fluxes on ER are defined as:
  10 $${D}_{C{a}^{2+}}({\bf{n}}.\nabla [C{a}_{e}^{2+}]){| }_{Cyt}={b}_{e}(-{J}_{I{P}_{3}R}-{J}_{RYR}+\frac{1}{\alpha }{J}_{SERCA})$$
  11 $${D}_{C{a}^{2+}}({\bf{n}}.\nabla [C{a}_{MERC}^{2+}]){| }_{MERC}={b}_{e}(-{J}_{I{P}_{3}R,MERC}+\frac{1}{\alpha }{J}_{SERCA,MERC})$$
  in which b_*e*_ is the buffering capacity of ER. While the endoplasmic reticulum is a sprawling network of interconnected membranes containing a wide range of proteins and ribosomes, we simplify this network into a series of tubes that span the length of the model dendritic shaft.
- **Mito-ER Contacts:** The ER is known to closely contact the mitochondria and form junctions known as mitocondria-ER Contacts^19,53^. This close proximity, which can be as small as 10 nm and is filled with actin^82^, calcium binding proteins^20^, and pathways that inhibit calcium reuptake^53^. Therefore we model the calcium dynamics in these regions as a separate microdomain from the cytosol. In order to activate IP_3_R on the ER surface facing this microdomain, IP_3_ must also be present. Thus there are 2 main components within this microdomain, Ca^2+^ and IP_3_. We do not model the molecular composition of the MERC microdomain that control the influx and efflux of Ca^2+^. Rather, we use the diffusive fluxes at the MERC boundaries as lumped parameters to reflect the different protein distributions across the MERC boundaries. We model the geometry of this microdomain as a shell that surrounds the circumference of the mitochondria and connects to the endoplasmic reticulum. This shape was chosen to reflect EM images of MERCs and to efficiently model the portion of ER in contact with the mitochondria. The calcium concentration within this compartment is given by:
  12 $$\frac{\partial [C{a}_{MERC}^{2+}]}{\partial t}={D}_{C{a}^{2+}}{\nabla }^{2}[C{a}_{MERC}^{2+}]$$
  And the boundary conditions for the interface between the MERC and the ER, Mitochondria, and cytosol are given as follows:
  13 $${D}_{C{a}^{2+}}({\bf{n}}.\nabla [C{a}_{MERC}^{2+}]){| }_{Cyt}={b}_{e}({J}_{Ca,Diff})$$
  14 $${D}_{C{a}^{2+}}({\bf{n}}.\nabla [C{a}_{MERC}^{2+}]){| }_{ER}={b}_{e}(-{J}_{I{P}_{3}R,MERC}+\frac{1}{\alpha }{J}_{SERCA,MERC})$$
  15 $${D}_{C{a}^{2+}}({\bf{n}}.\nabla [C{a}_{MERC}^{2+}]){| }_{Mito}={b}_{m}(-{J}_{MCU,MERC}+{J}_{NCX,MERC})$$

#### Energetic considerations in the model

In this model, we focus on the impact on calcium on mitochondrial ATP production, as done in^27^. Our metabolic model focuses on NAD and NADH within the mitochondria, and ATP and ADP in both the mitochondria and cytosol. To avoid modeling the complex spatial patterns of the inner mitochondrial membrane, we assume that ADP conversion into ATP is a volumetric reaction within the mitochondria. Redox and mitochondrial potential are likewise modeled as volumetric terms within the mitochondria.

16 $$\frac{\partial [AT{P}_{m}]}{\partial t}={D}_{ATP}{\nabla }^{2}[AT{P}_{m}]+{J}_{F1FO}$$

ATP and ADP are exchanged through the Adenine Nucleotide Transporter (ANT), which is modeled as a boundary condition.

17 $${J}_{ANT}={V}_{ANT}\frac{(1-{R}_{c}{R}_{m}{e}^{-\frac{F{{\Delta }}\Psi }{RT}})}{(1+{R}_{c}{e}^{-f\frac{F{{\Delta }}\Psi }{RT}})(1+{R}_{m})}$$

After transfer into the cytosol, ATP freely diffuses and is hydrolyzed by unspecified reactions in the cytosol and modeled using the flux *J*_*HYD*_.

### Numerical methods

The Ca^2+^ concentrations and other variables concentrations in all compartments are high enough to be modeled through a deterministic approach. Simulations were conducted using the commercially available finite-element software COMSOL Multiphysics 5.4^83^. In order to solve our system of partial differential equations, we used time-dependent general partial differential equations and general boundary partial differential equations modules^83^. Starting with a coarse and unstructured mesh, we decreased the mesh size until we obtained the same results when using the maximum mesh size. COMSOL was allowed to optimize the element sizes through the "physics-controlled mesh" option. The linear system was solved directly by using the PARADISO solver on a Linux-based compute cluster. Newton’s method (nonlinear method) was used to linearize the system. Time integration was performed using a backward differentiation formula (BDF) with both adaptive order and adaptive step sizes. All COMSOL source files will be available on Rangamani lab Github page upon publication.

## Supplementary information

Supplementary Information

## Data Availability

All data files used to generate the figures in the paper are available on https://github.com/aleung15/MERC.

## Code availability

All computational modeling files used to generate the data in the paper are available on https://github.com/aleung15/MERC.
