# Supplementary material for: Systems modeling predicts that mitochondria ER contact sites regulate the postsynaptic energy landscape
Source: NPJ Syst Biol Appl. 2021 Jun 2;7:26. doi: 10.1038/s41540-021-00185-7 (PMC8172538; doi:10.1038/s41540-021-00185-7)
Supplement: Supplementary file 1 — Supplementary Information [file 41540_2021_185_MOESM1_ESM.pdf]

Supplementary Material

Supplementary Tables

Supplemental Table 1: Model geometry

Organelle	Shape	Length (μm)	Diameter (μm)	Surface area (μm^2)	Volume (μm^3)
Dendrite	cylinder	1.6	0.6		
Mitochondrion	cylinder	0.6	0.3		
Tubule mitochondrion	cylinder	1.2	0.21		
Each of 4 tubule ER	cylinder	1.5	0.08		
Spine 1 (head)	sphere	-	0.6		0.058 [43]
Spine 1 (neck)	cylinder	0.4	0.2		
Spine 1 (PSD)	spherical cap			0.132 [44]	
Spine 2 (head)	sphere	-	0.4		0.028 [43]
Spine 2 (neck)	cylinder	0.5	0.1		
Spine 2 (PSD)	spherical cap			0.055 [44]	
Spine apparatus (head)	sphere	-	0.34		
Spine apparatus (neck)	cylinder	0.5	0.06		
Spine apparatus (base)	cylinder	1.5	0.08		
Distance between spine 1 and spine 2	-	1	-		
Distance between the ER and mitochondria	-	0.050 [47]	-		
	-	0.030 [84]	-		

Supplemental Table 2: Fluxes used in the model and their description

Flux Equation	Involved Species	Reference
**Module 1:** Stimulus and Receptors		
ligand(glutamate)-receptor (mGluR) binding rate		
$J_{LR} = -k_b R_2 G^2 + k_u DIM$	R_2, G, DIM	[41]
PKC-mediated phosphorylation and phosphatase dephosphorylation of mGluR dimers		
$J_{DIM} = V_P \frac{DIM_p}{K_{mP}+DIM_p} - V_{PKC} \frac{PKC \cdot DIM}{K_{mPKC}+DIM}$	DIM, PKC	[41]
phosphorylated mGluR dimers		
$DIM_p = \frac{R_{tot}-\sqrt{K_{dim} R_2}-2R_2-2DIM}{2}$	R_2, DIM	[41]
Change in DAG-sensitive fraction of active PKC		
$\frac{dPKC}{dt} = k_{aPKC} \frac{DAG}{K_{mDAG}+DAG}(1-PKC) - k_{dPKC} PKC$	DAG, PKC	[41]
IP_3 synthesis by PLC		
$J_{IP_3} = k_{PLC_I} DIM$	DIM, IP_3	[41] [1]
DAG synthesis by PLC and DAG degradation by lipase and kinase		
$J_{DAG} = k_{PLC_D} DIM - V_{DAG} \frac{DAG}{K_{mDAG}+DAG}$	DIM, DAG	[41]
Ca^{2+} influx from NMDA receptor‡		
$J_{NMDA} = g_{NMDA} NMDA_O \frac{1}{1+(e^{0.092V})*0.28}$		[81]
$V = -65 + BPAP + EPSP$		
$BPAP = 0.04(0.75 \exp \frac{t-t_{bpf}}{\tau_{bf}} + 0.25 \exp \frac{t-t_{bps}}{\tau_{bs}})$		
$EPSP = 0.01(0.5 \exp -\frac{t}{\tau_{ef}} + 0.5 \exp -\frac{t}{\tau_{es}})$		

Flux Equation	Involved Species	Reference
**Module 2:** Calcium Dynamics		
Ca^{2+} current from the NMDA receptor (assuming $V_reversal$=0)		
$I_{Ca^{2+}} = \frac{-4(P_{Ca}/P_M)([Ca^{2+}_{ext}]/[M^+])\gamma RT}{2F}$	Ca^{2+}	[85]
Ca^{2+} efflux from the cytosol to the extracellular medium		
$J_{eff} = k_{ex} Ca_c^{2+}$	Ca_c^{2+}	[85]
Ca^{2+} flux from the ER into the cytosol through the IP_3 receptor		
$J_{IP_3R} = (k_{IP}b + k_{IP} IPR_{act})(Ca_e^{2+} - Ca_c^{2+})$	Ca_c^{2+},Ca_e^{2+}	
Fraction of active IP_3 receptors		
$IPR_{act} = (1-R_i) \frac{IP_3^2}{K_{IP}^2+IP_3^2} \frac{Ca_c^{na}}{K_{da}^{na}+Ca_c^{na}}$	Ca_c^{2+}	[29]
Change in the fraction of inactive IP_3 receptors		
$\frac{dR_i}{dt} = k_{ai}(1-R_i) \frac{Ca_c^{ni}}{1+(\frac{Ca_c}{K_{da}})^{na}} - k_{di} R_i$	Ca_c^{2+}	[29]
Ca^{2+} flux from the ER into the cytosol through the Ryanodine receptor		
$J_{RYR} = (V_{RYR} P_O + V_{leak})(Ca_e^{2+} - Ca_c^{2+})$	Ca_c^{2+}, Ca_e^{2+}	[86]
Open probability of Ryanodine receptor on the slow (second) timescale		
$P_O = \frac{\omega(1+Ca_c^3/K_b^3)}{K_a^4/Ca_c^4+1+Ca_c^3/K_b^3}$	Ca_c^{2+}	[86]
Fraction of channels not in Ca^{2+}-independent closed state ($\omega = 1 - P_{C_2}$)		
$\tau \frac{dw}{dt} = \omega_\infty - \omega$	Ca_c^{2+}	[86]
Equilibrium value of ω and it's relaxation time		
$\omega_\infty = \frac{1+K_a^4/Ca_c^4+Ca_c^3/K_b^3}{1+(1/K_c)+K_a^4/Ca_c^4+Ca_c^3/K_b^3} \quad \tau = \frac{\omega_\infty}{K_d}$	Ca_c^{2+}	[86]
Ca^{2+} flux from the cytosol into the ER through the SERCA ATPase pumps		

Continued on next page

Supplemental Table 2 – continued from previous page

Flux Equation	Involved Species	Reference
$J_{SERCA} = V_{SERCA} \frac{Ca_c^2}{K_p^2+Ca_c^2} \frac{ATP_c}{K_{ds}+ATP_c}$	Ca_c^{2+}, ATP_c	[97]
Ca^{2+} flux from the cytosol into the mitochondrion through the MCU channel		
$J_{MCU} = V_{MCU} \frac{\frac{Ca_c}{K_{trans}}(1+\frac{Ca_c}{K_{trans}})^3}{(1+\frac{Ca_c}{K_{trans}})^4+\frac{L}{(1+\frac{Ca_c}{K_{act}})^{nau}}} e^{p_1 \Delta\Psi}$	Ca_c^{2+}, $\Delta\Psi$	[97]
Ca^{2+} flux from the mitochondrion into the cytosol through the NCX		
$J_{NCX} = V_{NCX}(\frac{Ca_m}{Ca_c})e^{p_2 \Delta\Psi}$	Ca_c^{2+}, Ca_m^{2+}, $\Delta\Psi$	[27]
Ca^{2+} bidirectional flux of the mitochondrial Permeability Transition Pore		
$J_{mPTP} = k_{mPTP}(Ca_c - Ca_m)e^{p_3 \Delta\Psi}$	Ca_c^{2+}, Ca_m^{2+}, $\Delta\Psi$	[97]
Buffering Equation		
$J_{Buff} = k_{Buff}(Ca_c B - k_{eq,Buff} BCa)$	Ca_C, B, BCa	[26]
**Module 3:** Energetics		
Glycolytic pathway, Krebs cycle, and Pyruvate dehydrogenase (PDH)-catalysed reaction rate		
$J_{PDH} = k_{GLY}(\frac{Ca_m}{K_{mCa}+Ca_m})(\frac{1}{K_{mNAD}+\frac{NADH}{NAD^+}})$	Ca_m^{2+}, NADH, NAD^+	[27]
Ca^{2+} regulation of the Ca^{2+} binding mitochondrial Aspartate/glutamate carrier (AGC's)		
$J_{AGC} = V_{AGC}(\frac{Ca_c}{K_{AGC}+Ca_c})(\frac{K_{iCa}}{K_{iCa}+Ca_m})$	Ca_c^{2+}, Ca_m^{2+}	[97]
NADH oxidation in the Electron Transport Chain (ETC) and proton extrusion from mitochondria		
$J_O = V_O(\frac{NADH}{K_O+NADH})\frac{1}{1+e^{\frac{\Delta\Psi-p_4}{p_5}}}$	NADH, $\Delta\Psi$	[27]
ANT activity rate		
$J_{ANT} = V_{ANT}\frac{(1-R_c R_m e^{-\frac{F\Delta\Psi}{RT}})}{(1+R_c e^{-f\frac{F\Delta\Psi}{RT}})(1+R_m)}$	R_c, R_m, $\Delta\Psi$	[30]
$R_c = \frac{\alpha_c ATP_c}{ADP_c}, R_m = \frac{ADP_m}{\alpha_m ATP_m}$	ATP_c, ADP_c, ADP_m, ATP_m	
ATP synthesis/ADP phosphorylation rate by the F1FO ATPases		
$J_{F1FO} = V_{F1FO}\frac{1}{1+e^{\frac{p_6-\Delta\Psi}{p_7}}}(\frac{K_{iATP}}{K_{iATP}+ATP_m})$	ATP_m, $\Delta\Psi$	[27]
ATP consumption rate linked with Ca^{2+} activity in the cytosol		
$J_{HYD} = k_{HYD}(\frac{ATP_c}{K_{mHYD}+ATP_c})$	ATP_c, Ca_c^{2+}	[97]
Proton leak across the inner mitochondrial membrane		
$J_{Hleak} = p_8 \Delta\Psi + p_9$	$\Delta\Psi$	[27]

707 ‡See [81] for NMDAR receptor dynamics.

Supplemental Table 3: Initial conditions, boundary conditions and partial differential equations

**Variable**<br>Partial Differential Equation	*Definition*<br>Boundary Condition	Diffusion Coefficient<br>Initial Concentration
**Ca_c^{2+}**	*Cytosolic Calcium*	
$\frac{\partial[Ca_c]}{\partial t} = D_{Ca}\nabla^2[Ca_c]$ -J_{Buff}	$D_{Ca}(\mathbf{n}.\nabla[Ca_c])\|_{Mit} = b_c(-\beta J_{MCU} + \beta J_{NCX} - \beta J_{mPTP})m_m$	$D_{Ca_c^{2+}}$=65 (μm^2/s)[87]
	$D_{Ca}(\mathbf{n}.\nabla[Ca_c])\|_{ER} = b_c(\alpha J_{IP_3R} + \alpha J_{RYR} - J_{SERCA} - J_{Refill})m_e$	Ca_{c0}^{2+}=0.1 (μM) [88]
	$D_{Ca}(\mathbf{n}.\nabla[Ca_c])\|_{PSD} = b_c(J_{NMDA} - J_{eff})m_p$	
	$D_{Ca}(\mathbf{n}.\nabla[Ca_c])\|_{MERC} = (J_{Diff,MERC})m_p$	
**Ca_m^{2+}**	*Mitochondrial Calcium*	
$\frac{\partial[Ca_m]}{\partial t} = D_{Ca}\nabla^2[Ca_m]$	$D_{Ca}(\mathbf{n}.\nabla[Ca_m])\|_{Cyt} = b_m(J_{MCU} - J_{NCX} + J_{mPTP})m_m$	$D_{Ca_m^{2+}}$=30 (μm^2/s) [89]
	$D_{Ca}(\mathbf{n}.\nabla[Ca_m])\|_{MERC} = b_m(J_{MCU,MERC} - J_{NCX,MERC} + J_{mPTP,MERC})m_m$	Ca_{m0}^{2+}=0.1 (μM)[88]
**Ca_e^{2+}**	*ER Calcium*	
$\frac{\partial[Ca_e]}{\partial t} = D_{Ca}\nabla^2[Ca_e]$	$D_{Ca}(\mathbf{n}.\nabla[Ca_e])\|_{Cyt} = b_e(-J_{IP_3R} - J_{RYR} + \frac{1}{\alpha}J_{SERCA} + J_{Refill})m_m$	$D_{Ca_e^{2+}}$=1 (μm^2/s)[87]
	$D_{Ca}(\mathbf{n}.\nabla[Ca_e])\|_{MERC} = b_e(-J_{IP_3R,MERC} + \frac{1}{\alpha}J_{SERCA,MERC})m_m$	Ca_{e0}^{2+}=200 (μM)[88]
**Ca_{MERC}^{2+}**	*Mitochondria-ER Contact Calcium*	
$\frac{\partial[Ca_{MERC}]}{\partial t} = D_{Ca}\nabla^2[Ca_e]$	$D_{Ca}(\mathbf{n}.\nabla[Ca_{MERC}])\|_{Cyt} = J_{Diff,MERC}$	$D_{Ca_{MERC}^{2+}}$=1 (μm^2/s)[87]
	$D_{Ca}(\mathbf{n}.\nabla[Ca_{MERC}])\|_{Mito} = b_m(J_{MCU,MERC} - J_{NCX,MERC} + J_{mPTP,MERC})m_m$	$Ca_{MERC_0}^{2+}$=200 (μM)[88]
	$D_{Ca}(\mathbf{n}.\nabla[Ca_{MERC}])\|_{ER} = b_e(-J_{IP_3R,MERC} + \frac{1}{\alpha}J_{SERCA,MERC})m_m$	
**ATP_c**	*Cytosolic ATP*	
$\frac{\partial[ATP_c]}{\partial t} = D_{ATP}\nabla^2[ATP_c] - J_{HYD}$	$D_{ATP}(\mathbf{n}.\nabla[ATP_c])\|_{Mit} = m_m J_{ANT}$	D_{ATP_c}=33 (μm^2/s)[90]
	$D_{ATP}(\mathbf{n}.\nabla[ATP_c])\|_{ER} = -J_{SERCA}/2$	$ATP_{c,0}$=1300 (μM)[97]
**ADP_c**	*Cytosolic ADP*	
$\frac{\partial[ADP_c]}{\partial t} = D_{ADP}\nabla^2[ADP_c] + J_{HYD}$	$D_{ADP}(\mathbf{n}.\nabla[ADP_c])\|_{Mit} = -m_m J_{ANT}$	D_{ADP_c}=30 (μm^2/s)[1]
	$D_{ADP}(n.\nabla[ADP_c])\|_{ER} = J_{SERCA}/2$	ADP_{c0}=1200 (μM)[97]
**IP_3**	*Second Messenger IP_3*	
$\frac{\partial[IP_3]}{\partial t} = D_{IP_3}\nabla^2[IP_3] - k_{deg}(IP_3 - IP_{3,0})$	$D_{IP_3}(\mathbf{n}.\nabla[IP_3])\|_{PSD} = J_{IP_3}$	D_{IP_3}=283 (μm^2/s)[91]
		$IP3_0$=0.2 (μM) [92]
**R_i**	*Fraction of Ca^{2+}-inhibited IP_3Rs*	
		R_{i0}=0.9898 [92]

**R_2**<br>$\frac{\partial[R_2]}{\partial t} = D_{R_2}\nabla^2[R_2] + J_{LR}$	*Free mGluR dimers*	D_{R_2}=0.001 ($\mu m^2/s$)[1]<br>R_2=0.002 (μM) [41]
**DIM**<br>$\frac{\partial[DIM]}{\partial t} = D_{DIM}\nabla^2[DIM] + J_{DIM}$	*Active, nonphosphorylated mGluR dimers*<br>$D_{DIM}(\mathbf{n}.\nabla[DIM])\|_{PM} = -J_{LR}$	D_{DIM}=0.001 ($\mu m^2/s$)[1]<br>DIM_0=0 mol/m^2 [1]
**DIM_p**<br>$\frac{\partial[DIM_p]}{\partial t} = -D_{DIM_p}\nabla^2[DIM_p] + J_{DIM_p}$	*Active, phosphorylated mGluR dimers*	D_{DIM_p}=0.001 ($\mu m^2/s$)<br>$DIM_{P0} = 3 \times 10^{-12}\ mol/m^2$[1]
**DAG**<br>$\frac{\partial[DAG]}{\partial t} = D_{DAG}\nabla^2[DAG] + J_{DAG}$	*Second messenger DAG*	D_{DAG}=10 ($\mu m^2/s$)[93]<br>DAG_0=0.025 (μM) [92]
**PKC**	*Fraction of active protein kinase C*	PKC_0=0.2 [92]
**NADH**<br>$\frac{\partial[NADH]}{\partial t} = D_{NADH}\nabla^2[NADH] + J_{PDH} - J_O$	*Mitochondrial NADH*<br>$D_{NADH}(\mathbf{n}.\nabla[NADH])\|_{Cyt} = (J_{AGC})m_m$	D_{NADH}=30 ($\mu m^2/s$)[94]<br>$NADH_0$=50 (μM)[97]
**NAD^+**<br>$\frac{\partial[NAD]}{\partial t} = D_{NAD}\nabla^2[NAD] - J_{PDH} + J_O$	*Mitochondrial NAD^+*<br>$D_{NAD}(\mathbf{n}.\nabla[NAD])\|_{Cyt} = (-J_{AGC})m_m$	D_{NAD}=30 ($\mu m^2/s$)[94]<br>NAD_0=200 (μM)[97]
**ADP_m**<br>$\frac{\partial[ADP_m]}{\partial t} = D_{ADP}\nabla^2[ADP_m] - J_{F1FO}$	*Mitochondrial ADP*<br>$D_{ADP}(\mathbf{n}.\nabla[ADP_m])\|_{Cyt} = (J_{ANT})m_m$	D_{ADP_m}=30 ($\mu m^2/s$) [84]<br>ADP_{m_0}=5000 (μM)[97]
**ATP_m**<br>$\frac{\partial[ATP_m]}{\partial t} = D_{ATP}\nabla^2[ATP_m] + J_{F1FO}$	*Mitochondrial ATP*<br>$D_{ATP}(\mathbf{n}.\nabla[ATP_m])\|_{Cyt} = (-J_{ANT})m_m$	D_{ATP_m}=30 ($\mu m^2/s$) [84]<br>ATP_{m_0}=10000 (μM)[97]
**$\Delta\Psi$**<br>$\frac{\partial(\Delta\Psi)}{\partial t} = D_e\nabla^2(\Delta\Psi)$	*Voltage difference across the inner mito membrane*<br>$-D_e(\mathbf{n}.\nabla(\Delta\Psi))\|_{Mit} = (a_1 J_O - a_2 J_{F1FO})/C_p$<br>$D_e(\mathbf{n}.\nabla(\Delta\Psi))\|_{Cyt} = (-J_{Hleak} - J_{NCX} - 2J_{MCU}$<br>$-2J_{mPTP} - J_{ANT} - J_{AGC})/C_p$	$D_{\Delta\|Psi}$=2000 ($\mu m^2/s$)[1]<br>$\Delta\Psi_0$=150 (mV) [96]

NMDA Receptor Cascade	*Glutamatergic NMDAR activation cascade* [81]	
$\frac{dN_{C0}}{dt} = -R_b * N_{C0} * Glut + R_u * N_{C1}$		$N_{C0,0} = 1$
$\frac{dN_{C1}}{dt} = R_b * N_{C0} * Glut - R_u * N_{C1}$		
$\quad -R_b * N_{C1} * Glut + R_u * N_{C2}$		$N_{C1,0} = 0$
$\frac{dN_{C2}}{dt} = R_b * N_{C1} * Glut - R_u * N_{C2}$		
$\quad -(R_o * N_{C2} - R_c * N_O) - (Rd * N_C2 - Rr * N_D)$		$N_{C2,0} = 0$
$\frac{dN_O}{dt} = (R_o * N_{C2} - R_c * N_O)$		$N_{O,0} = 0$
$\frac{dN_D}{dt} = (Rd * N_C2 - Rr * N_D)$		$N_{D,0} = 0$

[1] This Work

Supplemental Table 4: Model Parameters

Flux	Parameter	Definition	Value	Units	Reference
	b_c	buffering capacity of cytosol	0.01	-	
	b_m	buffering capacity of mitochondria	0.01	-	
	b_e	buffering capacity of ER	0.0003	-	
	m_p	cytosol volume to postsynaptic surface area ratio	9.3032×10^{-8}	m	1
	m_m	cytosol volume to mitochondrion surface area ratio	6.004×10^{-8}	m	1
	m_e	cytosol volume to ER surface area ratio	2.615×10^{-7}	m	1
	α	ER volume to cytosol volume ratio	0.099814		1
	β	mitochondria volume to cytosol volume ratio	0.05		1
	κ_i	endogenous buffer capacity	20		[85]
J_{NMDA}	R_b	Glutamate Binding Rate	5	$s^{-1}\mu M^{-1}$	[81]
J_{NMDA}	R_u	Glutamate Unbinding Rate	12.9	s^{-1}	[81]
J_{NMDA}	R_d	NMDAR Desensitization	8.4	s^{-1}	[81]
J_{NMDA}	R_o	NMDAR Opening	46.5	s^{-1}	[81]
J_{NMDA}	R_r	NMDAR Recycling	6.8	s^{-1}	[81]
J_{NMDA}	g_{NMDA}	Conductivity	0.6	nS	[81]
J_{NMDA}	Mg^{2+}	Magnesium Concentration	1	mM	[81]
J_{NMDA}	E	Reversal Potential	0	V	[81]
J_{NMDA}	t_{bpf}	BPAP fast delay time	0.002	s	[81]
J_{NMDA}	t_{bps}	BPAP slow delay time s	0.002	s	[81]
J_{NMDA}	τ_{bf}	BPAP fast time constant	0.025	s	[81]
J_{NMDA}	τ_{bs}	BPAP slow time constant	0.003	s	[81]
J_{NMDA}	τ_{ef}	EPSP fast time constant	0.005	s	[81]
J_{NMDA}	τ_{es}	EPSP slow time constant	0.05	s	[81]
J_{eff}	k_{ex}	Ca^{2+} clearance rate constant in the absence of Ca^{2+} buffer	0.0016	s^{-1}	[85]
J_{LR}	k_u	kinetic constant of glutamate unbinding to mGluR dimers	2	s^{-1}	[41]
J_{LR}	k_b	kinetic constant of glutamate binding to mGluR dimers	1	$\mu\mathrm{M}^{-2}\mathrm{s}^{-1}$	[41]
J_{DIM}	V_P	maximal rate of mGluR dephosphorylation by a phosphatase	0.05	$\mu\mathrm{Ms}^{-1}$	[41]
J_{DIM}	K_{mP}	Michaelis-Menten constant of mGluR dephosphorylation by a phosphatase	5×10^{-4}	μM	[41]
J_{DIM}	V_{PKC}	maximal rate of mGluR phosphorylation by PKC	0.2	$\mu\mathrm{Ms}^{-1}$	[41]
J_{DIM}	K_{mPKC}	Michaelis-Menten constant of mGluR phosphorylation by PKC	5×10^{-4}	μM	[41]
J_{DIM}	K_{dim}	dimerization constant of mGluR	0.1	μM	[41]
J_{DIM}	R_{tot}	total concentration of mGluR in monomeric form	0.075	μM	[41]
J_{DIM}	k_{aPKC}	Normalized (divided by PKC total conc.) maximal rate of PKC activation	0.2	s^{-1}	[41]
J_{DIM}	k_{dPKC}	rate constant of PKC deactivation	0.2	s^{-1}	[41]
J_{DIM}	K_{mDAG}	activation constant of PKC by DAG	0.06	μM	[41]
J_{IP_3}	k_{PLC_I}	catalytic activity of PLC for IP_3	1.25	s^{-1}	[41]
J_{IP_3}	k_{DAG_I}	DAG enhancing IP_3 production via its positive feedback effect on PLC activity	8	s^{-1}	1
J_{IP_3}	k_{dI}	rate constant of IP_3 degradation (to IP_2 and IP_4)	0.12	s^{-1}	[41]
J_{DAG}	k_{PLC_D}	catalytic activity of PLC for DAG	1.25	s^{-1}	[41]
J_{DAG}	V_{DAG}	maximal rate of DAG degradation	0.0325	$\mu\mathrm{Ms}^{-1}$	[41]
J_{DAG}	K_{DAG}	Michaelis-Menten constant of DAG degradation	0.012	μM	[41]

Continued on next page

Supplemental Table 4 – continued from previous page

Flux	Parameter	Definition	Value	Units	Reference
J_{RYR}	V_{RYR}	rate constant of the Ca^{2+} flux from the ER into the cytosol through the RYR	5	s^{-1}	[86]
J_{RYR}	V_{leak}	basal flux in the absence of RYR receptors/ rate of Ca^{2+} leak from the ER?	0.15	s^{-1}	[86]
J_{RYR}	K_a^4	RYR dissociation constant	0.019	μM^4	[86]
J_{RYR}	K_b^3	RYR dissociation constant	0.257	μM^3	[86]
J_{RYR}	K_c	RYR dissociation constant	0.057	-	[86]
J_{RYR}	K_d	rate constant for transition between open and closed state	0.1	s^{-1}	[86]
J_{IP_3R}	k_{IP}	rate constant of the Ca^{2+} flux from the ER into the cytosol through the IP_3R	30	s^{-1}	[29]
J_{IP_3R}	$k_{IP}b$	basal flux in the absence of IP_3 receptors/ rate of Ca^{2+} leak from the ER	0.3	s^{-1}	[29]
J_{IP_3R}	K_{IP}	dissociation constant of IP_3 binding to IP_3 receptor	1	μM	[29]
J_{IP_3R}	K_{da}	dissociation constant of Ca^{2+} binding to the activating site of IP_3 receptor	0.3	μM	[29]
J_{IP_3R}	n_a	Hill coefficient for cooperative Ca^{2+}-binding of IP_3 receptor activation	3	-	[29]
J_{IP_3R}	n_i	Hill coefficient for cooperative Ca^{2+}-binding of IP_3 receptor inhibition	4	-	[29]
J_{IP_3R}	k_{ai}	association constant of Ca^{2+}-binding to the inhibitory site of IP_3 receptor	20	$\mu M^4.s^{-1}$	[29]
J_{IP_3R}	k_{di}	dissociation constant of Ca^{2+}-binding to the inhibitory site of IP_3 receptor	0.02	s^{-1}	[29]
J_{SERCA}	V_{SERCA}	maximal rate of the SERCA pumps	120	$\mu M.s^{-1}$	[29]
J_{SERCA}	K_p	Ca^{2+} dissociation constant from the SERCA pumps	0.35	μM	[29]
J_{SERCA}	K_{ds}	ATP dissociation constant from the SERCA pumps	0.05	μM	[29]
$J_{IP3MERC,cyt}$	$K_{diff,IP3}$	Diffusive flux rate between MERC and cytoplasm	10	$1/s$	[1]
$J_{CaMERC,cyt}$	$K_{diff,CA}$	Diffusive flux rate between MERC and cytoplasm	0.25	$1/s$	[1]
J_{MCU}	V_{MCU}	maximal rate of the mitochondrial Ca^{2+} uniporter	0.0006	$\mu M.s^{-1}$	[85]
J_{MCU}	K_{trans}	dissociation constant of Ca^{2+} transport in uniporter	25	μM	[85]
J_{MCU}	K_{act}	dissociation constant of uniporter activation by Ca^{2+}	6	$\mu M.$	[85]
J_{MCU}	L	allosteric constant for transition between relaxed and taut form in four equivalent binding domains of the uniporter	50	-	[85]
J_{MCU}	n_{au}	uniporter activation cooperativity parameter	2.3	-	[85]
J_{MCU}	p_1	uniporter voltage dependence parameter	0.1	mV^{-1}	[85]
J_{NCX}	V_{NCX}	maximal rate of the NCX	0.35	μM^{-1}	[85]
J_{NCX}	p_2	exchanger voltage dependence parameter	0.016	mV^{-1}	[85]
J_{mPTP}	k_{PTP}	rate constant of the bidirectional Ca^{2+} flux through the mPTP	0.008	s^{-1}	[29]
J_{mPTP}	p_3	bidirectional Ca^{2+} leak voltage dependence parameter	0.05	mV^{-1}	[29]
J_{PDH}	k_{GLY}	Glyceraldehyde 3-phosphate dehydrogenase (GPDH) reaction rate	450	$\mu M.s^{-1}$	[29]
J_{PDH}	K_{mCa}	Michaelis-Menten constant for Krebs cycle activation by mitochondrial Ca^{2+}	0.1	μM	[29]
J_{PDH}	K_{mNADH}	Michaelis-Menten constant for NAD^+ consumption in the Krebs cycle	1	μM	[29]
J_{AGC}	V_{AGC}	maximal rate of NADH production via Malate/asparate shuttle (MAS)	25	$\mu M.s^{-1}$	[29]
J_{AGC}	K_{AGC}	cytosolic Ca^{2+} dissociation constant from the AGC	0.14	μM	[29]
J_{AGC}	K_{iCa}	inhibition constant of AGS by cytosolic Ca^{2+}	0.3	μM	[98-99]
J_O	V_O	maximal rate of NADH oxidation in the ETC	600	$\mu M.s^{-1}$	[29]
J_O	K_O	Michaelis-Menten constant for NADH oxidation in the ETC	1000	μM	[29][1]
J_O	p_4	ETC voltage dependence parameter 1	177	mV	[29]
J_O	p_5	ETC voltage dependence parameter 2	5	mV	[29]
J_{ANT}	V_{ANT}	maximal rate for ADP_c/ADP_m exchange in the absence of ATP	5	$mM.s^{-1}$	[29]
J_{ANT}	α_c	cytosolic ADP and ATP buffering parameter	0.111	-	[29]

Continued on next page

Supplemental Table 4 – continued from previous page

Flux	Parameter	Definition	Value	Units	Reference
J_{ANT}	α_m	mitochondrial ADP and ATP buffering parameter	0.139	-	[29]
J_{ANT}	f	fraction of $\Delta\Psi$ responsible for the large increase in the Michaelis-Menten constant for ATP uptake by energized mitochondria	0.5	-	[29]
J_{F1FO}	V_{F1FO}	maximal rate of F1FO ATPase	35	$mM.s^{-1}$	[29]
J_{F1FO}	p_6	F1FO ATPase voltage dependence parameter 1	200	mV	[29][1]
J_{F1FO}	p_7	F1FO ATPase voltage dependence parameter 2	8.5	mV	[29]
J_{F1FO}	K_{iATP}	F1FO ATPase inhibition constant by mitochondrial ATP	10000	μM	[29][1]
J_{HYD}	k_{HYD}	maximal rate of ATP hydrolysis	80	$\mu M.s^{-1}$	[51]
J_{HYD}	K_{mHYD}	Michaelis-Menten constant for ATP hydrolysis	1000	μM	[29]
J_{Hleak}	p_8	proton leak voltage dependence parameter	2	$\mu M.s^{-1}.mV^{-1}$	[29]
J_{Hleak}	p_9	proton leak pH dependence constant	-30	$\mu M.s^{-1}$	[29]
J_{Refill}	K_{Refill}	Refill Rate of ER from Cytoplasm (Non-SERCA)	0.035	$1/s$	[1]

1 This Work

Supplementary Figures

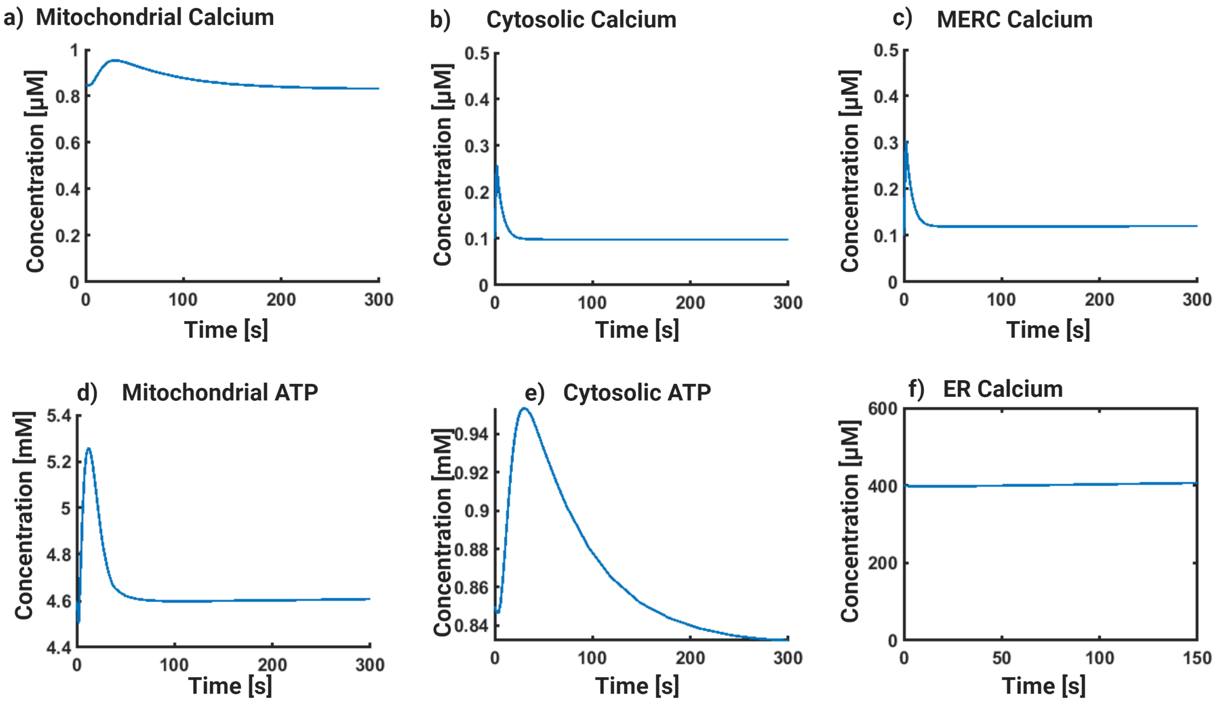

Supplemental Figure 1: **Simulations without glutamate response quickly decay toward steady-state. a**) Mitochondrial calcium dynamics with no stimulus for MERC SA ratio of 0.1 mitochondria surface area and mitochondria sized 0.6 μm. **b**) Same as **a**) for Cytosolic Calcium. **c**) Same as **a**) for MERC Calcium. **d**) Same as **a**) for mitochondrial ATP. **e**) Same as **a**) for cytosolic ATO **f**) same as **a**, but for ER Calcium.

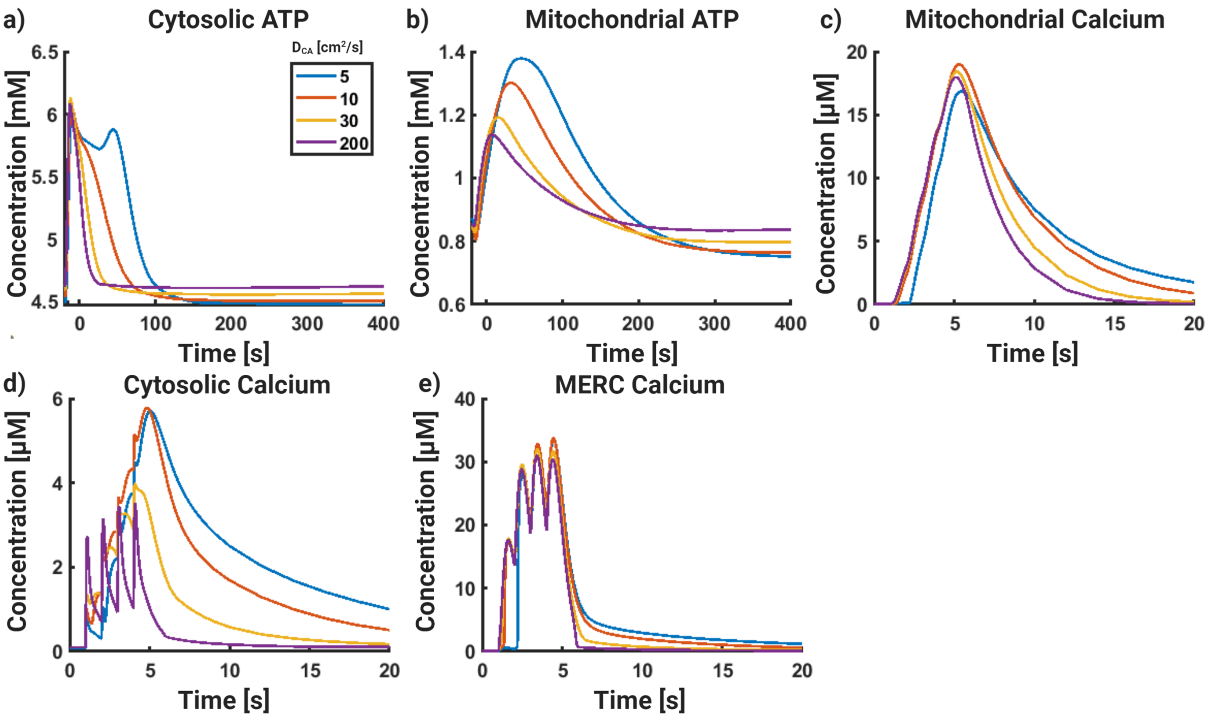

Supplemental Figure 2: **Calcium diffusion coefficient exhibits changes in system timescales a**) Cytosolic ATP dynamics in reponse to 5 1Hz pulses with varying calcium diffusion constants, 5,10,30, and 200 cm^2/s. **b**) Mitochondria ATP dynamics with varying calcium diffusion constants. **c**) Mitochondrial dynamics with varying diffusion constants of free calcium. **d**) Cytosolic calcium dynamics with varying calcium diffusion constants. **e**) MERC calcium dynamics with varying calcium diffusion constants.

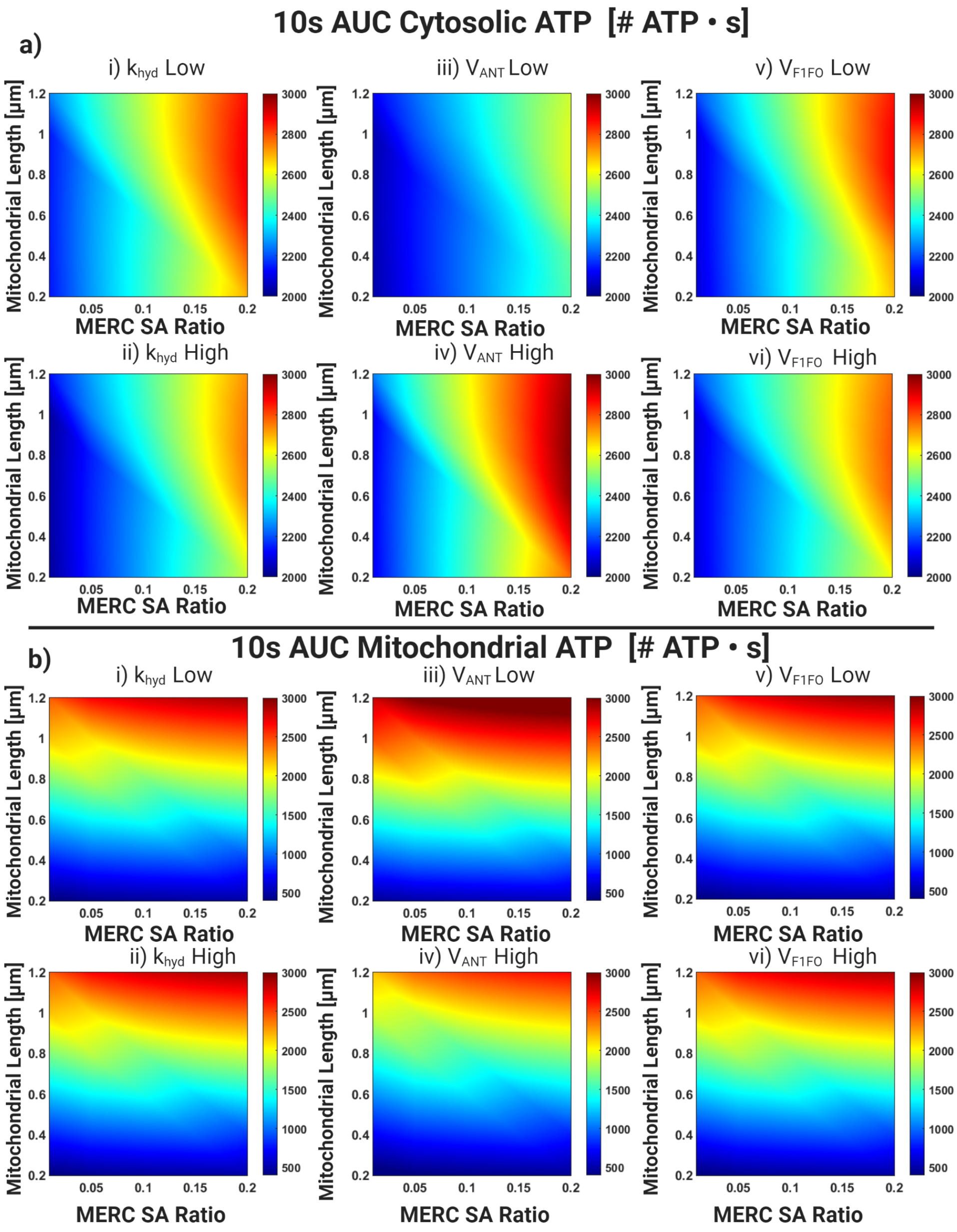

Supplemental Figure 3: **Phase Diagram for AUC of ATP for 10 seconds post-stimulus with varying metabolic parameters.** **a**) Cytosolic ATP AUC phase map for **i**) $k_{HYD} = 50\mu M/s$, **ii)** $k_{HYD} = 70\mu M/s$, **iii)** $V_{ANT} = 35mM/s$, **iv**) $V_{ANT} = 65mM/s$, **v**) $V_{F1FO} = 25mM/s$, **vi)** $V_{F1Fo} = 45mM/s$. **b**) Same as **a**) for mitochondria ATP.

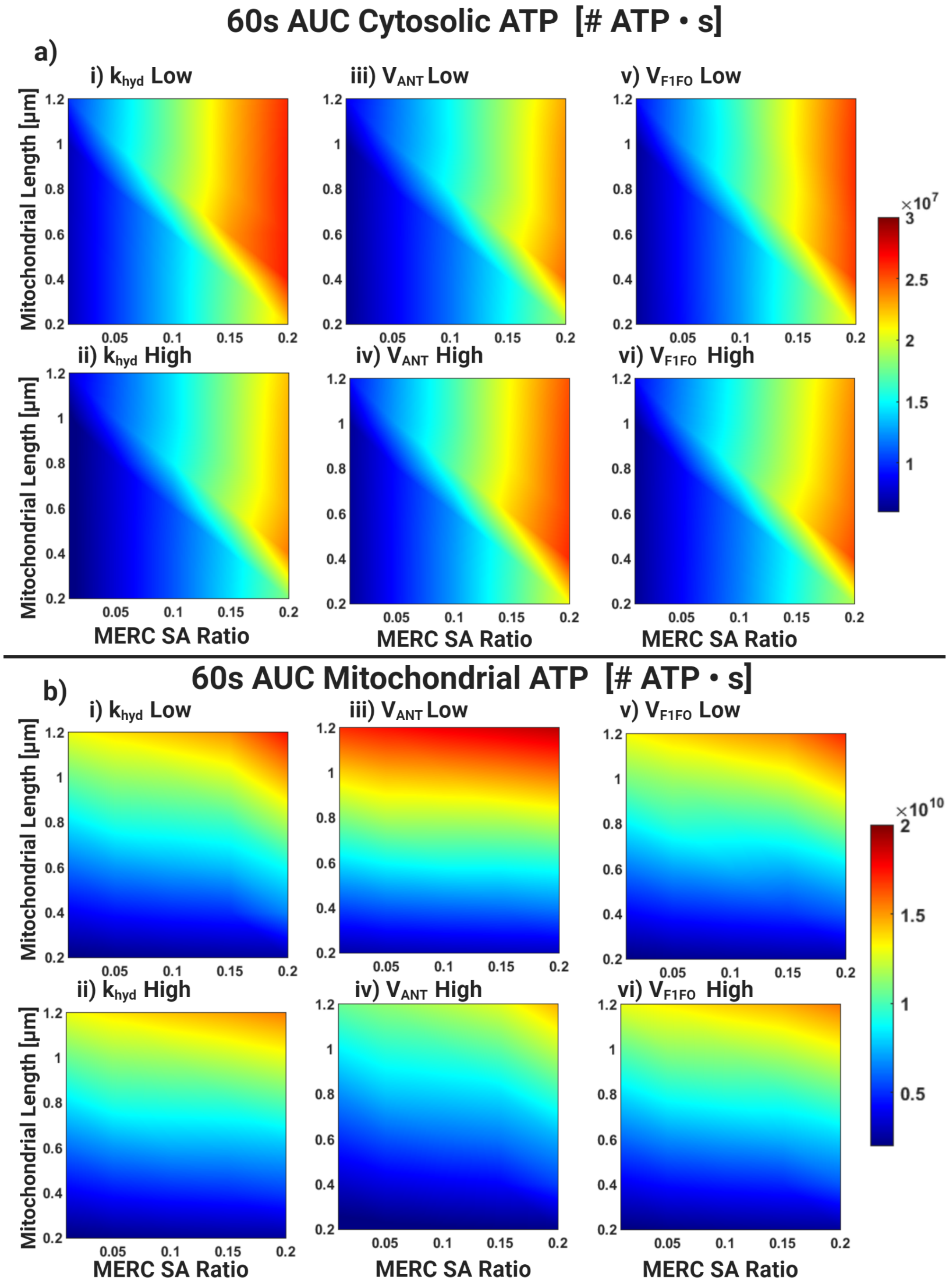

Supplemental Figure 4: **Phase Diagram for AUC of ATP for 60 seconds post-stimulus with varying metabolic parameters.** **a**) Cytosolic ATP AUC phase map for **i**) $k_{HYD} = 50\mu M/s$, **ii)** $k_{HYD} = 70\mu M/s$, **iii)** $V_{ANT} = 35mM/s$, **iv)** $V_{ANT} = 65mM/s$, **v)** $V_{F1FO} = 25mM/s$, **vi)** $V_{F1Fo} = 45mM/s$. **b**) Same as **a**) for mitochondria ATP.

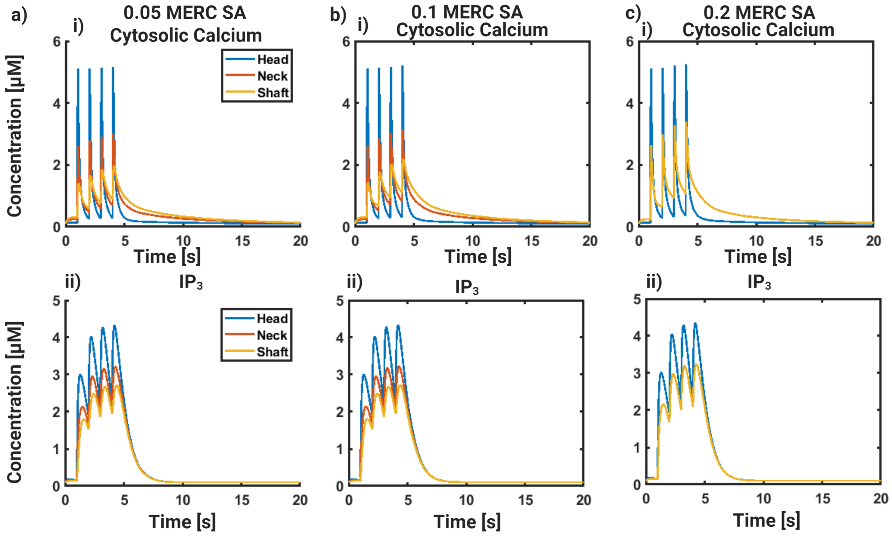

Supplemental Figure 5: **Point graphs for Calcium and IP$_3$ dynamics with respect to changing MERC SA Fraction a**) **i**) Calcium dynamics at cytosol in spine head, neck, and shaft for system with 0.6 μm mitochondria and 0.05 MERC SA fraction. **ii)** Corresponding IP$_3$ dynamics . **b**) Same as **a**for 0.1 MERC SA fraction. **c**) Same as **c**for 0.2 MERC SA fraction.

Supplemental References

[84] Lombardi, A. A. & Elrod, J. W. Mediating ER-mitochondrial cross-talk. Science 358, 591–592 (2017).

[85] Nimchinsky, E. A. The number of glutamate receptors opened by synaptic stimulation in single hippocampal spines. J. Neurosci. 24, 2054–2064 (2004).

[86] Keizer, J. & Levine, L. Ryanodine receptor adaptation and Ca2+(-)induced Ca2+ release-dependent Ca2+ oscillations. Biophys. J. 71, 3477–3487 (1996).

[87] Yasuda, R. Biophysics of biochemical signaling in dendritic spines: implications in synaptic plasticity. Biophys. J. 113, 2152–2159 (2017).

[88] Szabadkai, G. & Duchen, M. R. Mitochondria: the hub of cellular Ca 2+ signaling. Physiology 23, 84–94 (2008).

[89] Greget, R. et al. Simulation of postsynaptic glutamate receptors reveals critical features of glutamatergic transmission. PLoS ONE 6, e28380 (2011).

[90] Rostovtseva, T. K. & Bezrukov, S. M. ATP transport through a single mitochondrial channel, VDAC, studied by current fluctuation analysis. Biophys. J. 74, 2365–2373 (1998).

[91] Decrock, E. et al. IP3, a small molecule with a powerful message. Biochim. Biophys. Acta 1833, 1772–1786 (2013).

[92] Manninen, T., Havela, R. & Linne, M.-L. Reproducibility and comparability of computational models for astrocyte calcium excitability. Front. Neuroinf. 11, 11 (2017).

[93] Brown, S.-A., Morgan, F., Watras, J. & Loew, L. M. Analysis of Phosphatidylinositol-4,5-bisphosphate signaling in cerebellar purkinje spines. Biophys. J. 95, 1795–1812 (2008).

[94] Zare, H. R. & Golabi, S. M. Caffeic acid modified glassy carbon electrode for electrocatalytic oxidation of reduced nicotinamide adenine dinucleotide (NADH). J. Solid State Electrochem. 4, 87–94 (2000).

[95] Bowen, W. J. & Martin, H. L. The diffusion of adenosine triphosphate through aqueous solutions. Arch. Biochem. Biophys. 107, 30–36 (1964).

[96] Nicholls, D. G., Vesce, S., Kirk, L. & Chalmers, S. Interactions between mitochondrial bioenergetics and cytoplasmic calcium in cultured cerebellar granule cells. Cell Calcium 34, 407–424 (2003).

[97] Wacquier, B., Romero Campos, H. E., González-Vélez, V., Combettes, L. & Dupont, G. Mitochondrial Ca 2+ dynamics in cells and suspensions. FEBS J. 284, 4128–4142 (2017).

[98] Rueda, C. B. et al. Ca2+ regulation of mitochondrial function in neurons. Biochim. Biophys. Acta 1837, 1617–1624 (2014).

[99] Contreras, L. et al. Ca2+ activation kinetics of the two aspartate-glutamate mitochondrial carriers, aralar and citrin. J. Biol. Chem. 282, 7098–7106 (2007).
